# Foliar infections by *Botrytis cinerea* modulate the tomato root volatilome and microbiome

**DOI:** 10.1093/femsec/fiaf042

**Published:** 2025-04-18

**Authors:** Muhammad Syamsu Rizaludin, Ana Shein Lee Díaz, Hans Zweers, Jos M Raaijmakers, Paolina Garbeva

**Affiliations:** Department of Microbial Ecology, The Netherlands Institute of Ecology (NIOO-KNAW), Droevendaalsesteeg 10, 6708 PB Wageningen, The Netherlands; Department of Microbial Ecology, The Netherlands Institute of Ecology (NIOO-KNAW), Droevendaalsesteeg 10, 6708 PB Wageningen, The Netherlands; Department of Microbial Ecology, The Netherlands Institute of Ecology (NIOO-KNAW), Droevendaalsesteeg 10, 6708 PB Wageningen, The Netherlands; Department of Microbial Ecology, The Netherlands Institute of Ecology (NIOO-KNAW), Droevendaalsesteeg 10, 6708 PB Wageningen, The Netherlands; Institute of Biology, Leiden University, Slyviusweg, 2333 BE Leiden, The Netherlands; Department of Microbial Ecology, The Netherlands Institute of Ecology (NIOO-KNAW), Droevendaalsesteeg 10, 6708 PB Wageningen, The Netherlands

**Keywords:** foliar infection, plant systemic response, root microbiome, root volatilome

## Abstract

The fungal pathogen *Botrytis cinerea* causes significant damage to aboveground plant parts, but its impact on root chemistry and microbiome composition is less understood. This study investigated how *B. cinerea* foliar infection influences the root volatilome and microbiome of two tomato genotypes: wild *Solanum pimpinellifolium* and domesticated *Solanum lycopersicum* var. Moneymaker. In the absence of infection, wild tomato roots emitted higher levels of monoterpenes such as α-pinene and terpinene compared to domesticated tomato roots. The fungal infection induced elevated levels of benzyl alcohol and benzofuran in the root headspace and/or rhizosphere of both genotypes, alongside genotype-specific changes. Multivariate analyses revealed that *B. cinerea* significantly altered bacterial and fungal community compositions in the rhizosphere and rhizoplane, with stronger bacterial community shifts in the rhizoplane. Taxa depletion and enrichment were observed, particularly among Proteobacteria and Ascomycota. Mantel tests showed significant correlations between rhizoplane bacterial community compositions and root-associated volatilome. Notably, enriched bacterial taxa such as *Pelomonas* and *Comamonadaceae* positively correlated with benzyl alcohol and benzofuran levels in the root volatilome. These findings demonstrate that *B. cinerea* foliar infection might induce profound changes in root-associated volatilome and microbiome composition, highlighting its systemic effects on plant root chemistry and microbiome composition.

## Teaser

*Botrytis cinerea* infection in tomato leaves triggers changes in root volatilome and microbiome, revealing connections between aboveground infections and underground plant interactions with their environment.

## Introduction

*Botrytis cinerea* is a foliar fungal pathogen that infects a wide range of crop plants leading to decay of leaves, stems, flowers, and fruits. While the direct effects of *B. cinerea* and other foliar fungal pathogens on the aerial parts of plants are well-documented, their impact on plant root chemistry and microbiome assembly remains largely unexplored. Foliar infections by fungal or bacterial pathogens can trigger systemic effects in plants, which primes the plant for defense against subsequent attacks (Berendsen et al. 2018, Luo et al. 2022). Interestingly, foliar stresses can trigger belowground plant responses by altering root exudate composition both quantitatively and qualitatively, which in turn selectively enriches or depletes specific root-associated microbial taxa leading to induction of resistance (Yi et al. 2011). For instance, when *Arabidopsis thaliana* was infected with the downy mildew pathogen *Hyaloperonospora arabidopsidis*, bacterial consortia were recruited, which induced resistance against the same foliar pathogen (Berendsen et al. 2018). Similarly, foliar infection of *Panax notoginseng* with *Alternaria panax* affected the composition and concentrations of root exudate constituents such as specific sugars and organic acids, which coincided with the enrichment of specific beneficial microbes in the rhizosphere and concomitant positive plant–soil feedback (Luo et al. 2022). *Botrytis cinerea* has been reported to induce systemic responses in tomato plants by enhancing root secretion of gluconic acid, which was associated with an enrichment of beneficial fungi in the rhizosphere (Fernández et al. 2017).

The objective of our study was to investigate how foliar infection of tomato leaves by *B. cinerea* influences the root and rhizosphere volatilome, as well as the composition of root-associated bacterial and fungal communities. Specifically, we examined whether pathogen-induced changes in volatile organic compounds (VOCs) emissions coincide with shifts in microbial community structure. We hypothesized that *B. cinerea* infection alters both root and rhizosphere volatile profiles, as well as the composition of the root-associated microbiome, and that these effects may depend on tomato genotype. We focused on VOCs as they can serve as important belowground chemical signals for short- and long-distance plant–microbe communications (Schulz-Bohm et al. 2018, Sharifi et al. 2022). Their unique chemical nature as small molecules with a low boiling point and high vapor pressure allows these compounds to diffuse through soil matrices and convey messages distances away from the emitters (Wenke et al. 2010). Soil microbes, such as bacteria and fungi, can perceive these volatiles and be attracted or repelled. Reciprocally, bacteria and fungi can emit VOCs, which may affect plant growth and resistance to environmental stresses (Weisskopf et al. 2021). To date, however, little is known about the impact of aboveground foliar stress on this chemical interplay.

To investigate the impact of *B. cinerea* on the tomato root volatilome and microbiome composition, we used two different tomato genotypes: *Solanum pimpinellifolium* and *Solanum lycopersicum* var. Moneymaker representing a wild and domesticated tomato, respectively. We investigated whether foliar infections of these tomato genotypes change the root volatilome and whether these changes coincide with specific changes in the composition of the root-associated bacterial and fungal communities. We developed nondestructive rhizosphere-root volatile sampling techniques using HiSorb traps to collect root VOCs in *in vitro* (sterile) and in greenhouse (nonsterile) assays. To achieve this, we conducted untargeted volatilome analyses using gas chromatography–mass spectrometry (GC/MS), along with 16S rRNA and internal transcribed spacer (ITS) gene amplicon sequencing, to characterize changes in tomato root chemistry and microbiome in response to foliar infection by *B. cinerea*.

## Materials and methods

### Soil collection and preparation

Soil samples were obtained from an organically managed agricultural plot in Nergena, Bennekom, The Netherlands, with coordinates 51.996250, 5.659375. Collected in 2014, the top 80-cm layer of soil was excavated from the field and then stored outdoors without intervention, allowing the natural soil ecosystem, including wild plants, to remain intact. The soil was air-dried, sieved through a 5-mm mesh to eliminate larger particles and plant debris, and subsequently stored in dark, room temperature conditions until further use.

### Seed sterilization and pregermination

Two tomato genotypes—*S. lycopersicum* var. Moneymaker (purchased from Bingenheimmer Saatgut AG, Echzell, Germany) and its wild counterpart *S. pimpinellifolium* (provided by Wageningen Seed Science Centre, Wageningen, The Netherlands)—were used. These genotypes have been extensively used in phenotyping studies, particularly regarding characteristics such as seed, seedling, and root traits (Khan et al. 2012, Kazmi et al. 2017). Tomato seeds from both genotypes were surface sterilized by immersion in 70% (v/v) ethanol for 2 min, and 1.5% (v/v) sodium hypochlorite for 15 min. Subsequently, the seeds underwent three rinses with ample volumes of sterile demineralized water. To ensure surface sterility, 100 µl of demineralized water from the final rinse was plated onto 1/10th strength tryptic soy agar and 1/2 strength potato dextrose agar to check for bacterial and fungal growth, respectively. For the greenhouse assay, the sterilized seeds were placed on sterile, premoistened filter paper inside sterile Petri dishes, and incubated at 25°C in the dark until germination.

### Fungal strain preparation

*Botrytis cinerea* strain B05.10 was kindly provided by Dr Jan van Kan from the Department of Phytopathology, Wageningen University and Research. The fungal strain was cultured on 1/2 strength malt extract agar (Difco) and incubated at 20°C in darkness for 14 days until sporulation. The fungal spores were harvested and adjusted to a concentration of 10^6^ spores/ml for the infection assay.

### Greenhouse experimental setup and plant growth conditions

Pregerminated tomato seeds were transplanted into PVC pots (Desch Plantpark, The Netherlands) with dimensions of 7 × 7 × 8 cm, containing 300 g of previously mentioned agricultural soil with an initial moisture content of 20% (v/w). A total of 10 pots were prepared for each tomato genotype for the collection of rhizosphere and rhizoplane samples, while 4 pots were left unplanted for the collection of bulk soil. Additionally, 5 additional pots were prepared per genotype to assess the disease severity index following *B. cinerea* infection. Tomato seedlings were cultivated inside a controlled greenhouse environment at 23°C with a 16-h light/8-h dark photoperiod and 80% humidity. Plants received 20 ml of 1/2 strength Hoagland solution and water weekly to maintain optimal growth. Upon reaching the fifth true leaf stage, 5 biological replicates of each tomato genotype were inoculated with *B. cinerea* by inoculating 10 µl of the spore suspension [diluted in half strength potato dextrose broth (PDB), 10^6^ spores/ml] onto the surface of tomato leaflets originating from the third and fourth true leaves. Foliar infection was allowed to develop for another 2 weeks, during which the leaves were re-inoculated weekly with the same concentration of spore solution to sustain the infection. Fungal infection was quantified by measuring the area of the lesions formed on the surface of inoculated leaflets. Five replicates of each genotype were mock-inoculated with 10 µl of sterile 1/2 strength PDB, representing the healthy control treatment.

### Fungal infection bioassay and disease scoring

Two weeks after fungal spore inoculations, tomato plants (including those in the healthy treatment) were harvested for measurement of shoot biomass (dry), and for collection of rhizosphere, rhizoplane, and bulk samples. Additionally, five additional plants for each genotype were harvested for the detached leaf bioassay. For this, leaflets originating from the fourth true tomato leaves were detached (five biological replicates and three technical replicates per genotype), immersed in 70% (v/v) ethanol for 5 min, washed with sterile demineralized water, and placed onto sterile filter paper inside a 9-cm Petri dish before being treated with 10 µl of spore solution (10^6^ spores/ml) or mock-inoculated with 1/2 strength PDB. The plates were then incubated at 23°C, and the lesions were allowed to develop for 4 days post-inoculation. WINFOLIA^TM^ software was utilized to determine the lesion area (discolored tissue), to quantify the percentage (%) of infected over the noninfected leaflet areas.

### Rhizosphere volatilome profiling *in planta*

For the collection of volatiles from the soil–root interface in the greenhouse assay, HiSorb probes (Markes International Ltd, Llantrisant, UK) were inserted into cylindrical metal holders previously planted ∼5 cm deep in the soil, closely positioned to the tomato root, to trap the root-associated volatilome (Supplementary Fig. S1). These metal holders, made of stainless steel with perforations around the surface, allow volatile diffusion into the cylinder while preventing direct contact between the probes and the soil, thereby creating a headspace. The top of the metal holders was equipped with screw caps enabling the cylinder to be closed when the probes were inside. The HiSorb probes were placed inside the metal holders overnight before recollection, 13 days after fungal spore application. The probes were also placed inside the holders to trap volatiles produced by the healthy control group, as well as the unplanted bulk soils. After recollection, HiSorb traps were placed into clean empty closed metal holders with screw caps at both ends (Markes International Ltd, Llantrisant, UK), and stored at room temperature until measurement.

### Root volatilome profiling *in vitro*

We designed an *in vitro* setup using two-compartment plates to profile root volatiles of two tomato genotypes exposed to *B. cinerea* under sterile conditions. This system ensured optimal trapping of only root volatiles and minimized potential contamination from shoot volatiles (Supplementary Fig. S2). In summary, surface-sterilized tomato seeds were placed inside tubes connected to one side of two-compartment Petri plates containing 20 ml of solidified 1/2 strength Murashige and Skoog (MS) medium. The other side of the compartment was left empty for the placement of the HiSorb probe. The plates were tightly sealed with parafilm, then placed inside a UV-sterilized box, and incubated in a climate chamber with a constant temperature of 23°C and a 16-h light/8-h dark photoperiod. Once the plants reached the third to fourth true leaves growth stage, two conditions were applied for each tomato genotype: the foliar stress with five biological replicates exposed to *B. cinerea* by inoculating 10 µl of a spore suspension in 0.5× potato dextrose broth (PDB, 10^6^ spores/ml) onto the surface of each leaflet from the third true leaves. The remaining five replicates were mock-inoculated using 10 µl of sterile 0.5× PDB, serving as the healthy control. Each set of replicates from the same treatment group was placed in one UV-sterilized box to prevent volatile cross-contamination. *Botrytis* infections were allowed to develop for 48 h before root volatile trapping.

### Volatile measurement using GC/Q-TOF

The volatiles were measured using gas chromatography coupled with quadrupole time-of-flight mass spectrometry (GC/Q-TOF) following the protocol outlined by Lee Diaz et al. (2022). In summary, VOCs were desorbed from HiSorb probes using an automated thermal desorption unit (Unity TD-100, Markes International, Llantrisant, UK) with helium gas at 240°C for 8 min. The released volatiles were then injected into the GC/Q-TOF system (Agilent 7890B GC and the Agilent 7200 Q-TOF, Santa Clara, CA, USA) with a DBms ultra-inert column (Agilent Technologies, Inc., Santa Clara, CA, USA). Mass fragments were detected by the GC/Q-TOF system operating at 70 eV in electron ionization mode with a source temperature of 230°C. A standard *n*-alkane mixture (C8–C20) was spiked at the beginning of the measurement to create a calibration curve. This curve was utilized to calculate the retention index (RI) of a compound based on its retention time (RT) relative to the known RT and RI of the alkane standard. The GC/Q-TOF raw data (.D) acquired at a full scanned mode (*m/z* 300–400, 4 scans/s, 2 GHz extended dynamic range).

### Volatile data analysis

The GC/Q-TOF raw data (.D) were first converted into content definition file (.cdf) format using GC AIA translator B.07.04 (Agilent Technologies, Santa Clara, CA, USA). Subsequently, the translated data were imported into Mzmine 2.53 (Pluskal et al. 2010) to extract a mass feature table. The Automated Data Analysis Pipeline algorithm (Du et al. 2020) was employed to perform chromatogram building, peak, and spectral deconvolution, as well as alignment steps (detailed parameters are available in the supplementary table). The mass feature table (.csv) was then subjected to filtering to eliminate features not biologically relevant including silica fragments prior to statistical analyses. The normalization of volatilome data was done using Metaboanalyst v6.0 (Pang et al. 2024). Briefly, the mass feature table was subject to normalization using log10 and Pareto-scalling. The principle component analysis was performed based on this normalized data. Permutational Multivariate Analysis of Variance (PERMANOVA) with 999 permutations was conducted to see whether there was any factor significantly affecting the volatile profile. Multiple pairwise comparison was then performed to obtain significantly different mass feature (*P* value < .05) between treatments using Metaboanalyst v6.0 for the normalized dataset. Heatmap was built to visualize the statistically significant mass features between treatments and clustered based on the Euclidean distance.

### Rhizosphere and rhizoplane sampling

Tomato plants, both healthy and *Botrytis*-infected, were uprooted from pots and roots were separated from the shoot. The roots were vigorously shaken to remove the loosely adhering soil. To collect rhizosphere and rhizoplane samples from the same root fragment, we modified a fractionation and detachment protocol previously described by Richter-Heitmann et al. (2016) and Attia et al. (2022). In brief, the roots with attached soil were placed into 50 ml Falcon tubes containing 35 ml of sterile 10 mM MgSO_4_ buffer. The solution was vortexed at maximum speed for 1 min, and the root was removed, leaving the soil suspension, which is considered as rhizosphere samples. The same roots were then transferred to new 50 ml Falcon tubes containing 35 ml of sterile 10 mM MgSO_4_ buffer, where the solution was subjected to 1 min vortex at maximum speed, followed by 1 min sonication. This process was repeated twice to further detach the remaining soil particles from the roots. After cleaning, the roots were removed, resulting in rhizoplane suspension. Bulk soil samples were collected from unplanted pots and then mixed directly with the same buffer as mentioned earlier. Bulk, rhizosphere, and rhizoplane suspensions were then centrifuged at 4500 rpm for 10 min to separate the soil pellet from the supernatant. The pellets were transferred to 2 ml tubes and then stored at −80°C until DNA extraction.

### DNA extraction, 16S and ITS-amplicon sequencing

Total DNA from different compartments (bulk, rhizosphere, and rhizoplane) was extracted using the DNeasy PowerSoil Pro kit (Qiagen, USA) according to the manufacturer protocol. The quantity and quality of the extracted DNA were checked using Nanodrop One (Thermo Fisher Scientific, USA) before sending the extract to BaseClear (BaseClear, B.V., Leiden, The Netherlands) for Illumina MiSeq sequencing. The primer set 341F (5′-CCTACGGGNGGCWGCAG-3′) and 785R (5′-GACTACHVGGGTATCTAATCC-3′) was used to amplify the V3–V4 region of the bacterial 16S rRNA gene. Meanwhile, the primer set of ITS1F (5′-TCCGTAGGTGAACCTGCGG-3′) and ITS2R (5′-GCTGCGTTCTTCATCGATGC-3′) was employed for amplification of the ITS1–ITS2 region of the fungal ITS. Each 25 µl polymerase chain reaction (PCR) reaction contained 12.5 µl of 2× KAPA HiFi HotStart ReadyMix, 5 µl of each primer (1 µM), and 2.5 µl of template DNA. Thermal cycling conditions included an initial denaturation at 95°C for 3 min; followed by 25 cycles of 95°C for 30 s, 55°C for 30 s, and 72°C for 30 s; and a final extension at 72°C for 5 min. The paired-end sequence reads (2 × 250 bp) generated by the Illumina MiSeq were then converted into FASTQ files using bcl2fastq (v2.20, Illumina) and were checked for quality with the FastQC tool (v0.12.0)

### Bioinformatic and statistical analyses

The demultiplexed paired-end files were processed to amplicon sequence variances (ASVs) using DADA2 pipeline 1.16 (Callahanetal.2016) run in R studio (version 2023.06.0+421). Before processing with the DADA2, primers were removed from the sequences using the Cutadapt function (v3.4). Sequencing error was checked and chimera were removed before assigning the reads for taxonomic identification using Silva (v1.38.1) and Unite database (v8.2) for bacteria and fungi, respectively. The resulting ASV abundance, taxonomy table, and the sample metadata were combined into a phyloseq object using phyloseq package (v1.42). Bacterial ASVs were firstly filtered out from affiliated with “eukaryote,” “archaea,” “mitochondria,” and “chloroplast.” To reduce noise and improve statistical power, the ASV table was further filtered to include only those with a minimum of 10 reads in at least four samples. α-Diversity, based on the Shannon index, was calculated on a rarefied ASV table (minimum read count of 5849) using the “get_alphaindex” function from the MicrobiotaProcess package (v1.10.3). A one-way analysis of variance (ANOVA) was performed to test the significance. Furthermore, β-diversity analysis was performed to assess differences in microbial composition between treatments. For this, principal coordinate analysis (PCoA) based on Aitchison distance was performed on the filtered ASVs [center-log ratio (clr)-transformed] using the microViz package (v0.10.8). PERMANOVA with 999 permutations was also conducted to investigate factors (compartment, genotype, and treatment) and significantly influenced microbial β-diversity. This was followed by a pairwise comparison using the pairwiseAdonis package (v0.4). To further investigate the difference in β-diversity of the two tomato genotypes under stress treatment without bulk soil effect, we repeated the previously mentioned analyses only on the ASVs enriched in the rhizosphere and rhizoplane. For this, a contrast test using the DESeq2 package (v1.38.3) was performed between bulk and rhizosphere, as well as between bulk and rhizoplane, to obtain ASVs enriched in the rhizosphere and rhizoplane, respectively [Benjamini–Hochberg with an adjusted p-value corresponding to a false discovery rate (FDR) < .05 and log-fold change (LFC) >2]. The same package was also used to conduct differential abundance analyses to determine ASVs significantly enriched or depleted upon *B. cinerea* infection of each of the two tomato genotypes corrected for the bulk soil effect.

The Mantel test based on Spearman’s correlation method with 999 permutations was conducted to investigate the association between fungal or bacterial communities and rhizosphere-associated volatilome. Spearman’s correlation analysis was further used to assess the correlation between differentially abundant ASVs and differential metabolites using the rcorr function from Hmsic package (v5.1–2). Significant correlations (*P* < .05*, *P* < .01**) were plotted in the heatmap using pheatmap package (v1.0.12).

## Results

### Susceptibility of tomato plants to *Botrytis cinerea*

Leaves of *S. lycopersicum* var. Moneymaker and *S. pimpinellifolium* were inoculated with spores of *B. cinerea*. Infection progressed more in the modern *S. lycopersicum* var. Moneymaker based on the higher percentage of lesion area compared to that of wild relative *S. pimpinellifolium* (Supplementary Fig. S3a). Furthermore, the dry shoot biomass of *Botrytis-*infected *S. lycopersicum* var. Moneymaker was substantially lower compared to the healthy plants, whereas in *S. pimpinellifolium*, the dry shoot biomass was slightly reduced in the infected plants compared to the noninfected (healthy) plants (Supplementary Fig. S3b).

### Impact of *Botrytis cinerea* infection on tomato rhizosphere and root volatilome

A total of 189 mass features corresponding to volatile compounds were detected from the five different treatments (unplanted bulk soil, healthy and *Botrytis*-infected *S. pimpinellifolium* and *S. lycopersicum* var. Moneymaker; Supplementary Data 1). PCoA indicated that the unplanted bulk soil clustered separately from the rest of the treatments suggesting that soil emitted a unique blend of volatiles (Supplementary Fig. S4a). We then filtered mass features that were significantly enriched relative to those detected in unplanted bulk soil (>2log-fold change, FDR < .05). In total, peak areas of 86 mass features were found to be significantly higher in the rhizosphere of the two tomato genotypes (with and without *B. cinerea* infection) compared to unplanted bulk soil (Supplementary Data 1).

**Figure 1.**
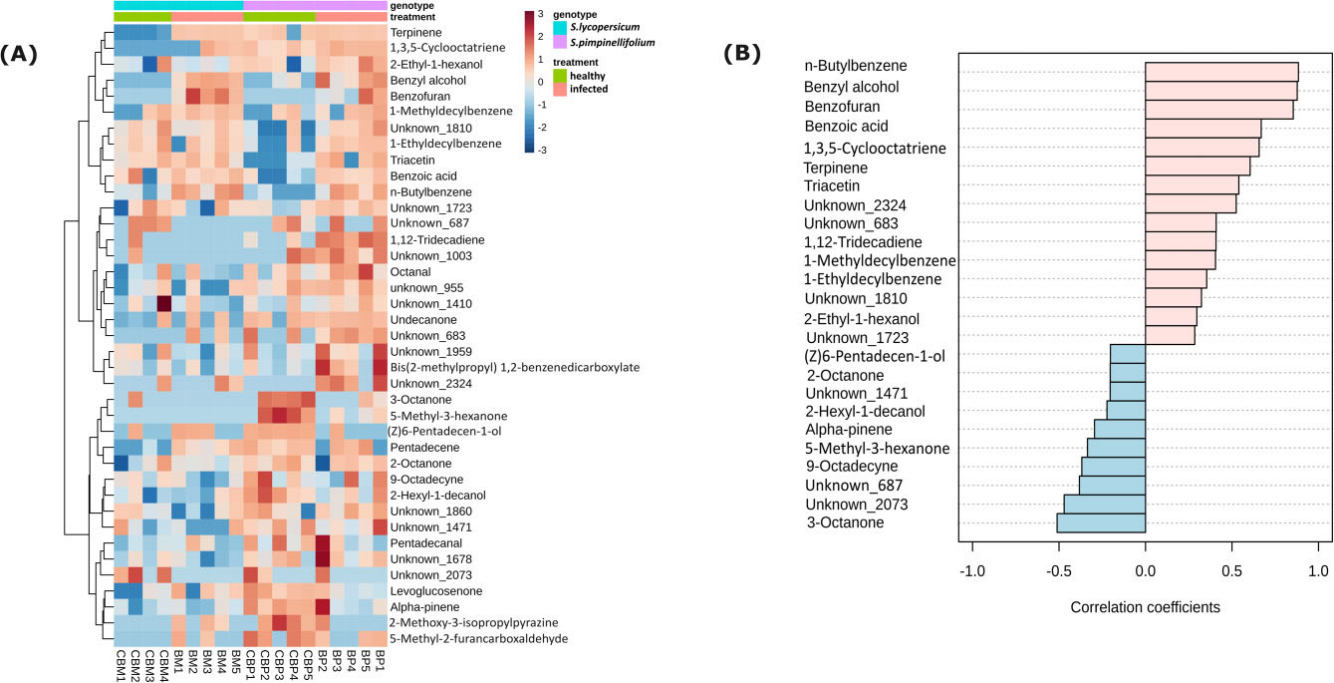
(A) Heatmap showing a comparison of normalized peak areas (log-transformed and Pareto-scaled) of selected mass features across different treatments. Mass features were selected based on their pairwise comparisons across treatments [healthy *S. lycopersicum* var. Moneymaker (CBM), healthy *S. pimpinellifolium* (CBP), Botrytis-infected *S. lycopersicum* var. Moneymaker (BM), and Botrytis-infected *S. pimpinellifolium* (BP)], using multiple testing correction (FDR). Only statistically significant features (FDR < .05) in at least one comparison are included in the heatmap. Clustering of the samples was performed using the Euclidean distance. The intensity of normalized peak area is indicated by the color gradient legend, with darker blue color suggesting a lower intensity while the darker red color indicating the higher peak area intensity. The bottom column codes refer to different treatments, with numbers referring to the number of replicate for each treatment. Some unidentified compounds are referred to unknowns followed by numbers indicating their calculated retention indices. (B) Spearman’s rank correlation coefficients of the top 25 root-associated volatile compounds differentially detected upon *B. cinerea* infection in tomato plants. Red bars and blue bars indicate compounds with positive and negative association with the infected plants, regardless of the genotype. *N*-butyl benzene, benzyl alcohol, and benzofuran are among the top three compounds positively associated with the stress responses of tomato plants (regardless of genotype) upon the infection of *B. cinerea*. Some unidentified compounds are referred to unknowns followed by numbers indicating their calculated retention indices.

Subsequent multivariate analysis for this filtered dataset showed that both the tomato genotype and treatment significantly affected the rhizosphere volatile profile (Supplementary Fig. S4a; Table 1). Tomato genotype explained the variation seen in the volatile profile more than the stress treatment (Table 1). In the absence of *Botrytis* infection (healthy plants), higher peak areas of monoterpenes such as α-pinene and terpinene were detected in the rhizosphere of wild *S. pimpinellifolium* than the modern *S. lycopersicum* var. Moneymaker (Supplementary Fig. S5a and b and Supplementary Data 2). Furthermore, we observed a significant interaction effect between the tomato genotype and stress treatment, suggesting that foliar infection by *B. cinerea* enhanced the emission of genotype-specific volatile compounds in the rhizosphere (Table 1). For infected *S. pimpinellifolium*, the volatile 1,2-tridecadiene, 1-ethyldecyl benzene, and triacetin were detected at higher abundance, whereas 3-octanone, 5-methyl 3-hexanone were detected at lower abundance compared to noninfected *S. pimpinellifolium* (Fig. 1A; Supplementary Fig. S6a and b and Supplementary Data 2). Infected *S. lycopersicum* showed reduced emission of (1-methyldecyl)benzene but an increase of terpinene emission (Supplementary Fig. S7a and b and Supplementary Data 2). Interestingly, the emission of benzyl alcohol, benzofuran, and *n*-butyl benzene was found to be higher in the rhizosphere of both tomato genotypes following *B. cinerea* leaf infections (Fig. 1A; Supplementary Figs S6 and S7 and Supplementary Data 2). This finding was in line with the results of the Spearman’s correlation coefficient showing that these volatile compounds were among the top three significant compounds discriminating the volatile profile of infected from that of noninfected tomato plants, regardless of the genotype (Fig. 1B).

**Table 1.** Pairwise PERMANOVA test based on the 999 permutations on the effect of tomato (genotype) and *B. cinerea* infection (treatment) and their interaction (genotype:treatment) on the profile of root-associated volatiles.^a^

	Rhizosphere volatilome	
PERMANOVA	*R* ^2^	*P* adjusted
Genotype	0.092	.01*
Treatment	0.077	.042*
Genotype:treatment	0.099	.004*
**Pairwise**		
CBP vs CBM	0.21	.048*
CBP vs BPP	0.21	.03*
CBM vs BM	0.177	.07
CBP vs BM	0.211	.03*
CBM vs BP	0.153	.192
BP vs BM	0.2	.03*

a In the pairwise panel, the following abbreviations apply: CBP, healthy *S. pimpinellifolium*; CBM, healthy *S. lycopersicum* var. Moneymaker; BP, Botrytis-infected *S. pimpinellifolium*; BM, Botrytis-infected *S. lycopersicum* var. Moneymaker.

* Statistical difference (FDR < .05).

To confirm whether some volatile compounds detected in the rhizosphere originated from the roots, we compared the volatile data from the greenhouse bioassays (nonsterile system) to our *in vitro* experiments (Supplementary Fig. S2), where we collected the root volatiles from two tomato genotypes (control and *Botrytis*-infected) under sterile conditions. Similar findings were also observed in our *in vitro* system where, in the absence of *B. cinerea* infection, the root volatile profile differs between two tomato genotypes, with higher peak areas of monoterpenes including α-pinene, β-pinene, terpinene, and limonene were detected in the roots of the wild tomato *S. pimpinellifolium* (Supplementary Fig. S8). Interestingly, despite system differences (different plant age and growing conditions), the stress-associated root volatiles detected in the greenhouse bioassays, in particular benzyl alcohol and benzofuran, were also detected *in vitro* for both tomato genotypes infected with *B. cinerea* (Supplementary Fig. S8a, b) suggesting that the same compounds were also induced in these genotypes following the infection of the same foliar pathogen.

### Impact of *Botrytis cinerea* leaf infection on root-associated bacterial community

A total of 3293 unique bacterial amplicon sequence variants (ASVs) were detected in bulk soil, rhizosphere, and rhizoplane of the two tomato genotypes. The bacterial α-diversity (Shannon index) was significantly lower in the rhizoplane compared to rhizosphere and bulk soil, whereas no significant differences in α-diversity were observed between bulk soil and rhizosphere (Supplementary Fig. S9a). The genotype and infection did not reveal any significant effects on the α-diversity for both rhizosphere and rhizoplane samples (Supplementary Fig. S9c). In contrast, principal coordinate analysis (PCoA) showed clustering based on the compartment: bulk soil, rhizosphere, and rhizoplane (Supplementary Fig. S10a). In general, the majority of bacterial ASVs belonged to the phyla *Proteobacteria* [currently known as *Pseudomonadota*], *Cyanobacteria, Actinobacteriota* [*Actinomycetota*], *Chloroflexi, Bacteroidota* [*Bacteroidetes*], and *Firmicutes* [*Bacillota*] (Supplementary Fig. S10c), differing in their relative abundances across the compartments (bulk soil, rhizosphere, and rhizoplane). The relative abundance of *Proteobacteria* [currently known as *Pseudomonadota*] gradually increased from bulk soil to rhizosphere and rhizoplane. Similarly, *Cyanobacteria* and *Bacteroidota* [*Bacteroidetes*] had higher relative abundances in the rhizoplane than in the rhizosphere and bulk soil. In contrast, the relative abundances of *Firmicutes* [*Bacillota*], *Actinobacteriota* [*Actinomycetota*], and *Chloroflexi* decreased in the rhizoplane (Supplementary Fig. S10c)

**Figure 2.**
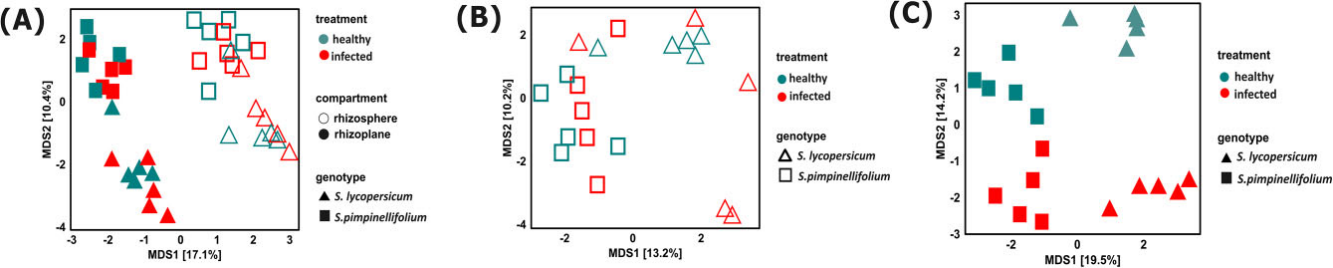
(A) Principal coordinate analysis (PCoA) based on Aitchison distance of bacterial community profile (of pooled data set) that were enriched in rhizosphere (empty shapes) and that of rhizoplane (empty shapes). The PCoA plot was then regrouped based on two compartments: rhizosphere (B) and rhizoplane (C). Each plot includes data for *S. lycopersicum* var. Moneymaker (indicated by triangle shapes) and *S. pimpinellifolium* (indicated by square shapes), with different colors representing different stress treatments; red for Botrytis-infected tomato plants (infected) and green for noninfected tomato plants (healthy).

To further investigate the impact of the foliar infection on the rhizosphere and rhizoplane bacterial community of the two tomato genotypes, we selected those bacterial ASVs that were significantly enriched in the rhizosphere and rhizoplane compared to that of bulk/unplanted soil samples (>2-fold change, adjusted *P* < .05). Multivariate analyses on this filtered dataset showed that the compartment (rhizosphere vs rhizoplane) remains explaining the biggest variations seen in the bacterial community diversity, followed by the tomato genotype, and stress treatment (Fig. 2A and Table 2). We therefore repeated the multivariate analyses for the rhizosphere and rhizoplane separately to look into the contribution of the genotype and stress factor to the bacterial community diversity in each compartment (Fig. 2B and C and Table 3). The bacterial community diversity was significantly influenced by tomato genotype for both rhizosphere and rhizoplane. The genotype factor better explained the variation seen in the β-diversity in the rhizoplane (∼17%) compared to the rhizosphere (∼10%). The foliar infection by *B. cinerea* had only a significant effect in shaping the bacterial community diversity in the rhizoplane, not in the rhizosphere.

**Table 2.** Pairwise PERMANOVA test based on the 999 permutations to investigate the contribution of root compartment (rhizosphere and rhizoplane), tomato genotype, and treatment (fungal infection) on bacterial and fungal community composition.

	Bacterial community		Fungal community	
PERMANOVA	*R* ^2^	*P* adjusted	*R* ^2^	*P* adjusted
Compartment	0.155	.001*	0.085	.001*
Genotype	0.093	.001*	0.048	.002*
Treatment	0.053	.003*	0.071	.001*

* Statistical difference (FDR < .05).

**Table 3.** Pairwise PERMANOVA test based on the 999 permutations on the effect of tomato genotype (genotype) and the fungal infection (treatment) as well as their interaction (genotype:treatment) on bacterial community diversity enriched in rhizosphere and rhizoplane.^a^

	16S rhizosphere		16S rhizoplane	
PERMANOVA	*R* ^2^	*P* adjusted	*R* ^2^	*P* adjusted
Genotype	0.102	.001*	0.175	.001*
Treatment	0.056	.169	0.133	.001*
Genotype:treatment	0.062	.07	0.084	.005*
**Pairwise**				
CBP vs CBM	0.186	.042*	0.324	.013*
CBP VS BP	0.129	.142	0.237	.013*
CBM VS BM	0.136	.145	0.289	.013*
CBP VS BM	0.194	.042*	0.358	.013*
CBM VS BP	0.145	.042*	0.316	.013*
BP VS BM	0.162	.058	0.274	.013*

a In the pairwise panel, the following abbreviations apply: CBP, healthy *S. pimpinellifolium*; CBM, healthy *S. lycopersicum* var. Moneymaker; BP, Botrytis-infected *S. pimpinellifolium*; BM, Botrytis-infected *S. lycopersicum* var. Moneymaker. *Statistical difference (FDR < .05).

### Differential abundances of bacterial taxa associated with *Botrytis* infection

To identify changes in the abundance of specific bacterial ASVs associated with the tomato genotype and foliar stress, we conducted a differential abundance analysis of all possible pairwise comparisons for rhizosphere and rhizoplane (Table 3). We found that rhizoplane bacterial communities showed larger changes in diversity than the rhizosphere indicated by more ASVs, which were differentially abundant (between treatments) in the rhizoplane than in the rhizosphere (Fig. 4A and B).

In general, foliar infection caused depletion and enrichment of several taxa in rhizoplane (Supplementary Data 3). Some of these changes were unique to each tomato genotype, while others were shared between the two. These changes were mainly associated with the phyla *Proteobacteria* (currently known as *Pseudomonadota*), *Actinobacteria* [*Actinomycetota*], and *Bacteroidota* [*Bacteroidetes*] (Fig. 4B; Supplementary Data 3). In *S. pimpinellifolium*, the foliar stress reduced the abundances of several ASVs belonging to the genera *Massilia* (ASV351, ASV367, ASV390, and ASV378), *Duganella* (ASV379, ASV317, and ASV678), *Devosia* (ASV186), *Burkholderia* (ASV455), *Flavobacterium* (ASV65), *Enterobacter* (ASV59), and *Actinoplanes* (ASV177). On the other hand, the relative abundances of *Cylindrospermum* (ASV61) and *Pantoea* (ASV135) were higher in the *Botrytis*-infected *S. pimpinellifolium*. For *S. lycopersicum* var. Moneymaker, we observed significantly lower abundances of *Flavobacterium* (ASV642), *Sphingomonas* (ASV454), *Stenotrophomonas* (ASV243), *Rahnella* (ASV33), and *Tychonema* (ASV78). Meanwhile, the relative abundances of *Leifsonia* (ASV776 and ASV493), *Nakamurella* (ASV496), *Pantoea* (ASV198), *Duganella* (ASV74 and ASV180), and *Pseudomonas* (ASV375) were higher in the rhizoplane of plants infected by *Botrytis*.

Interestingly, both *S. pimpinellifolium* and *S. lycopersicum* var. Moneymaker also showed similar patterns (Fig. 4B; Supplementary Tables S5 and S6) particularly for the lower abundances of ASVs belonging to *Enterobacteriaceae* (ASV6 and ASV9), *Pantoea* (ASV73), *Pseudomonas* (ASV119 and ASV259), *Burkholderia* (ASV455), and *Massilia* (ASV378). On the other hand, the rhizoplane of both tomato genotypes showed higher relative abundances of *Pelomonas* (ASV615), and an ASV from the *Comamonadaceae* (ASV261).

### Impact of *Botrytis* infections on root-associated fungal community

A total of 1968 unique fungal ASVs were identified by ITS sequencing of the bulk soil, rhizosphere, and rhizoplane samples of the two tomato genotypes. Unlike the bacterial community, the Shannon diversity index showed no statistically significant differences in fungal α-diversity between the three compartments (Supplementary Fig. S9b). Also, the fungal α-diversity was hardly influenced by the tomato genotype and foliar infection by *B. cinerea* (Supplementary Fig. S9b and d). The compartment factor (including bulk soil), however, significantly influenced the β-diversity as shown in the PCoA clustering (Supplementary Fig. 10b). In general, *Ascomycota, Basidiomycota*, and *Mortierellomycota* were among the most dominant phyla with slightly differing their abundances across compartments (Supplementary Fig. 10d).

**Figure 3.**
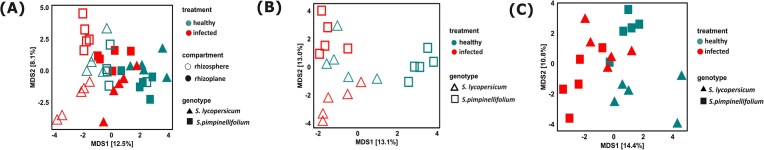
(A) Principal coordinate analysis (PCoA) based on Aitchison distance of fungal community profile (of pooled dataset) that were enriched in rhizosphere (empty shapes) and rhizoplane (filled shapes). The PCoA plot was then regrouped based on two compartments: rhizosphere (B) and rhizoplane (C). Each plot includes data for *S. lycopersicum* var. Moneymaker (indicated by triangle shapes) and *S. pimpinellifolium* (indicated by square shapes), with different colors representing treatment conditions: red for Botrytis-infected tomato plants (infected) and green for noninfected tomato plants (healthy).

We reconducted the multivariate analysis on the fungal ASVs that were only significantly enriched in rhizosphere and rhizoplane to normalize for the bulk soil effect (Fig. 3A). We found that compartment, tomato genotype, and stress treatment had significant effects on the fungal community diversity (Table 3). We then performed multivariate analysis for rhizosphere and rhizoplane separately to investigate the contribution of genotype and the foliar stress treatment on the β-diversity. Interestingly, both genotype and the stress factors significantly affected the fungal β-diversity for both rhizosphere and rhizoplane (Fig. 3B, C and Table 4). The influence of foliar stress on the fungal community, however, was slightly stronger in rhizoplane compared to that in the rhizosphere. Furthermore, the foliar stress treatment better explained the variation seen in the rhizoplane fungal community than the tomato genotype.

**Table 4.** Pairwise PERMANOVA test based on the 999 permutations on the effect of tomato genotype (genotype) and the fungal infection (treatment) as well as their interaction (genotype:treatment) on fungal community diversity enriched in rhizosphere (ITS rhizosphere) and rhizoplane (ITS rhizoplane).^a^

	ITS rhizosphere		ITS rhizoplane	
PERMANOVA	*R* ^2^	*P* adjusted	*R* ^2^	*P* adjusted
Genotype	0.088	.002*	0.083	.003*
Treatment	0.088	.002*	0.118	.001*
Genotype:treatment	0.104	.001*	0.093	.001*
**Pairwise**				
CBP vs CBM	0.190	.02*	0.206	.01*
CBP vs BP	0.236	.02*	0.237	.01*
CBM vs BM	0.189	.02*	0.224	.01*
CBP VS BM	0.245	.02*	0.221	.01*
CBM VS BP	0.132	.17	0.222	.01*
BP VS BM	0.231	.02*	0.193	.01*

a In the pairwise panel, the following abbreviations apply: CBP, healthy *S. pimpinellifolium*; CBM, healthy *S. lycopersicum* var. Moneymaker; BP, Botrytis-infected *S. pimpinellifolium;* BM; Botrytis-infected *S. lycopersicum* var. Moneymaker. *Statistical difference (FDR < .05).

### Differential abundances of fungal taxa associated with foliar stress

Differential abundant analysis was performed at the ASV level to identify changes between different treatments as indicated by the pairwise PERMANOVA (Table 4). We found that both rhizosphere and rhizoplane fungal communities exhibited similar numbers of differentially abundant ASVs across treatments (Fig. 5A and B). Changes in abundances of fungal ASVs to the stress treatment were different for the root compartments and tomato genotypes, but predominantly driven by taxa from Ascomycota (Supplementary Data 4 and 5). In the rhizosphere of *S. pimpinellifolium*, among other ASVs, *Purpureocillium* (ASV444), *Fusarium* (ASV61), *Mortierella* (ASV143), *Myrmecridium* (ASV186), and *Fusicola* (ASV219) were unique for the healthy plants but their abundances reduced following the foliar stress (Fig. 5A; Supplementary Data 4). Furthermore, ASVs belonging to the genus of *Trichoderma* (ASV73), *Olpidium* (ASV193), *Myceliophthora* (ASV259), and an unidentified ASV of the order Hypocreales (ASV248) were uniquely enriched in the infected *S. pimpinellifolium*.

In the rhizoplane of infected *S. pimpinellifolium* (Fig. 5B; Supplementary Data 5), ASVs with reduced abundances were noticeable such as *Chaetomium* (ASV39), *Setophoma* (ASV135), *Mortierella* (ASV42 and ASV153), and *Penicillium* (ASV191). On the other hand, the foliar infection increased the abundances of ASVs classified as *Annulohpoxylon* (ASV138), *Trichoderma* (ASV73), and an unidentified genus belonging to order Hypocreales (ASV248).

In the rhizosphere of *S. lycopersicum* var. Moneymaker, foliar stress specifically increased the abundances of *Monicillium* (ASV127 and ASV201), *Humicola* (ASV231), *Chaetomium* (ASV94), an ASV belonging to the phylum Rozellomycota, and three unidentified fungal ASVs (ASV59, ASV47, and ASV67). Meanwhile the abundances of *Fusarium* (ASV174), *Eucasphaeria* (ASV171), and *Acrostalagmus* (ASV93) decreased following the foliar stress (Fig 5A; Supplementary Data 4). In the rhizoplane of *S. lycopersicum* var. Moneymaker, the foliar stress increased the abundances of *Chaetomium* (ASV39), *Lachnum* (ASV128), *Acremonium* (ASV204), and few unidentified fungal ASVs (ASV67, ASV145, and ASV202) but decreased the abundances of *Fusarium* (ASV133) and *Acrostalagmus* (ASV93). Interestingly, few ASVs responded similarly for both tomato genotypes: higher relative abundances of *Trichoderma* (ASV73 and ASV74) and lower relative abundance of *Setophoma* (ASV135) were detected in the rhizoplane of both tomato genotypes following foliar infection by *Botrytis* (Fig 5B; Supplementary Tables S7 and S8).

### Correlations between changes in root-associated volatilome and microbiome

**Figure 4.**
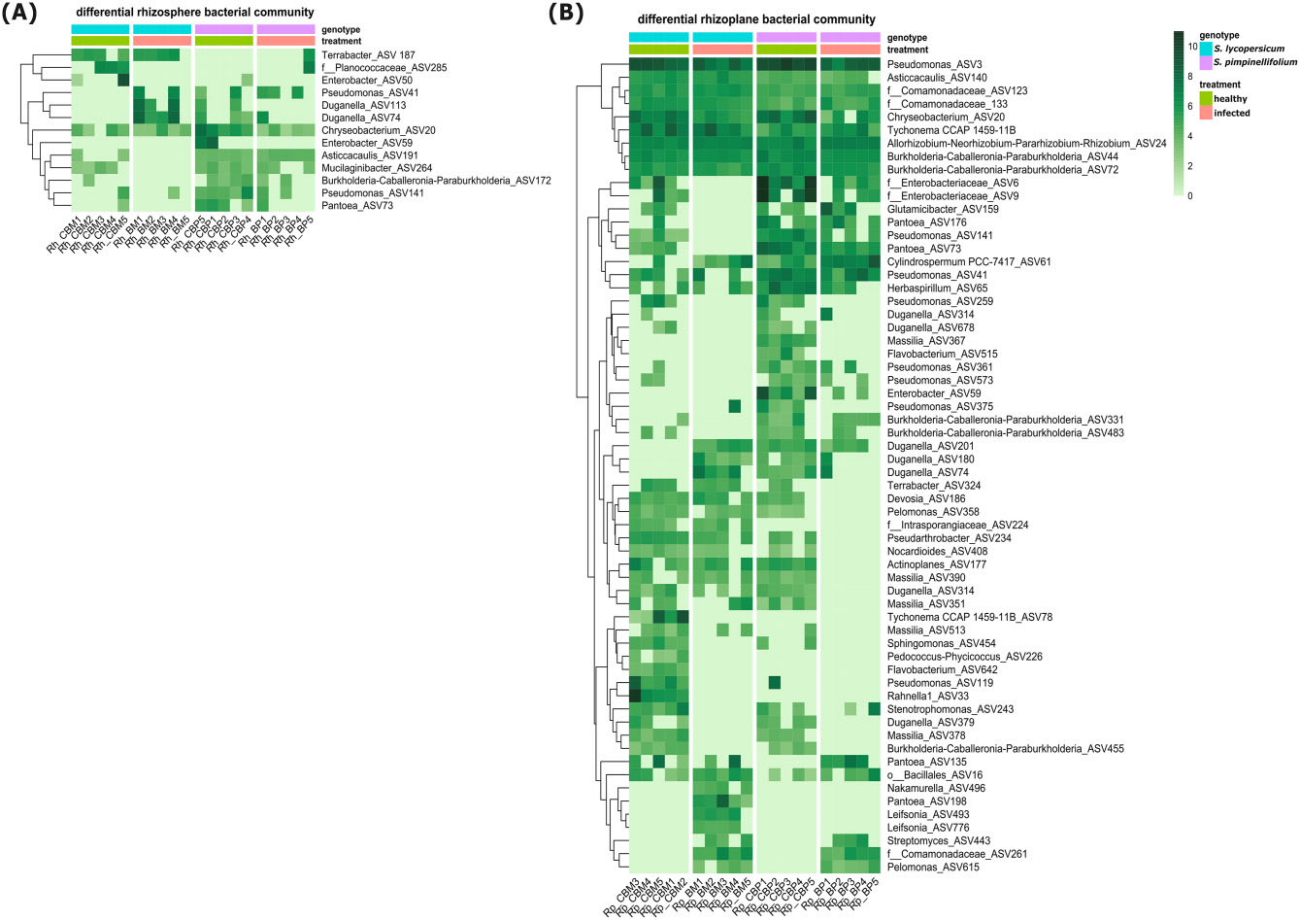
Heatmap showing the differentially abundant of bacterial amplicon sequence variants (ASVs) across treatments in the rhizosphere (A) and the rhizoplane (B) of *S. lycopersicum* var. Moneymaker and *S. pimpinellifolium* with and without *B. cinerea* infection (infected and healthy treatment, respectively). The differentially abundant taxa were selected based on the pairwise comparison across treatments [healthy *S. lycopersicum* (CBM), healthy *S. pimpinellifolium* (CBP), Botrytis-infected *S. lycopersicum* var. Moneymaker (BM), and Botrytis-infected *S. pimpinellifolium* (BP)], using Deseq2 package. ASVs with an adjusted *P*-value (FDR) < .05 were considered significantly differentially abundant. Only ASVs that were significantly differentially abundant in at least one comparison were included in the heatmap. Colors represents the value of log2(normalized count + 1). Bacterial taxa are represented by their genus names, unless unable to be identified; we refer to the closest taxonomy available such as order (symbolized by “o”) and family (symbolized by “f”) in front of the name. The row clustering is performed based on Euclidean distance. “Rh” and “Rp” in the beginning of codes in the heatmap column refer to rhizosphere and rhizoplane, respectively, whereas numbers at the end of code lines refer to the replicate number.

The Mantel test was performed to investigate whether changes in the root-associated and/or rhizosphere volatilome correlate with changes in the bacterial or fungal community composition in the rhizosphere and rhizoplane. Results revealed that only rhizoplane bacterial community had a significant correlation with the root-associated and/or rhizosphere volatilome (Table 5). In contrast, neither rhizoplane nor rhizosphere fungal communities showed statistically significant correlations with the root-associated and/or volatilome (Table 5). Subsequently, we performed Spearman’s correlation analysis to determine the association between individual bacterial taxa and a certain volatile compound. In general, we found significant positive and negative correlations of several bacterial taxa with root-associated volatiles (Fig. 6). Specifically, higher levels of volatile compounds associated with the stress response in tomato to *B. cinerea* (regardless of the genotype), particularly benzofuran and *n*-butyl benzene (Fig. 1B), negatively correlated with *Enterobacteriaceae* (ASV6), *Chryseobacterium* (ASV20), *Pantoea* (ASV73), *Pseudomonas* (ASV259), and *Massilia* (ASV378), bacterial taxa with relative abundances decreasing in the rhizoplane of both tomato genotypes upon foliar infection. In contrast, the abundance of these stress-associated volatile compounds such as benzyl alcohol, benzofuran, and *n*-butyl benzene positively correlated with the relative abundance of *Pelomonas* (ASV615) and an ASV from the *Comamonadaceae* (ASV261), bacterial taxa that are enriched in the rhizoplane of both tomato genotypes following foliar infection (Fig. 4B; Supplementary Fig. S11).

**Figure 5.**
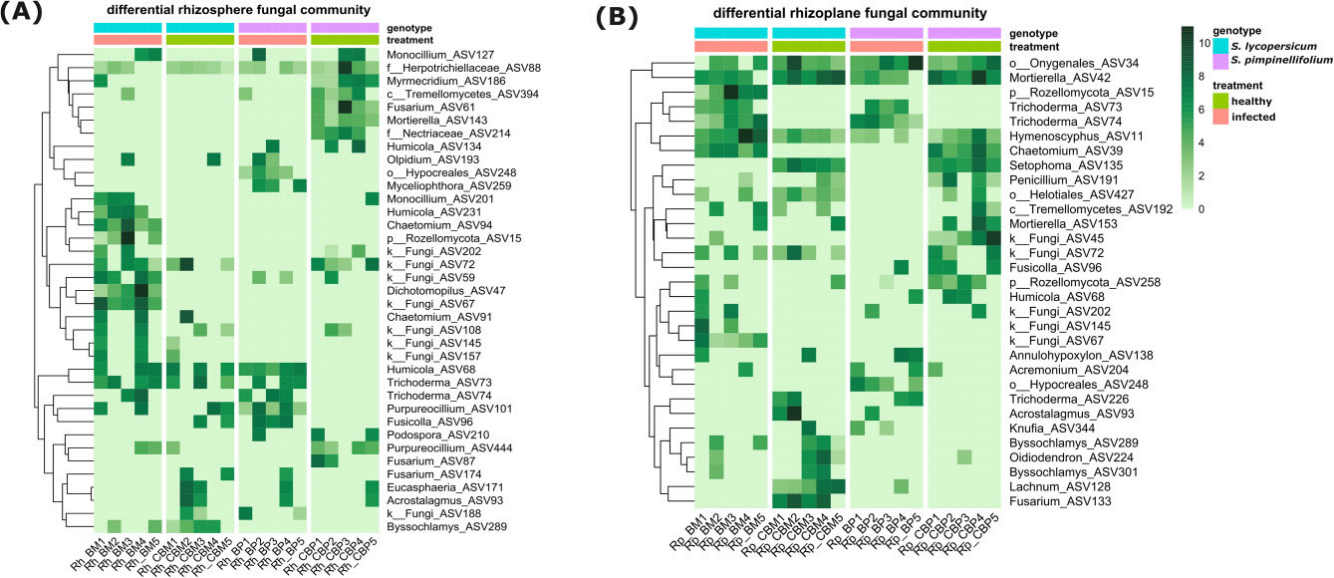
Heatmap showing the differentially abundant of fungal amplicon sequence variants (ASVs) across treatments in the rhizosphere (A) and the rhizoplane (B) of *S. lycopersicum* var. Moneymaker and *S. pimpinellifolium* with and without *B. cinerea* infection (infected and healthy treatment, respectively). The differentially abundant taxa were selected based on the pairwise comparison across treatments [healthy *S. lycopersicum* var. Moneymaker (CBM), healthy *S. pimpinellifolium* (CBP), Botrytis-infected *S. lycopersicum* var. Moneymaker (BM), and Botrytis-infected *S. pimpinellifolium* (BP)], using Deseq2 package. ASVs with an adjusted *P*-value (FDR) < .05 were considered significantly differentially abundant. Only ASVs that were significantly differentially abundant in at least one comparison were included in the heatmap. Colors represents the value of log2(normalized count + 1). Fungal taxa are represented by their genus names, unless unable to be identified; we refer to the closest taxonomy available such as kingdom (symbolized by “k”), order (symbolized by “o”), family (symbolized by “f”), and class (symbolized by “c”) in front of the name. The row clustering was performed based on Euclidean distance. “Rh” and “Rp” in the beginning of codes in the heatmap column refer to rhizosphere and rhizoplane, respectively, whereas numbers at the end of code lines refer to the replicate number.

**Figure 6.**
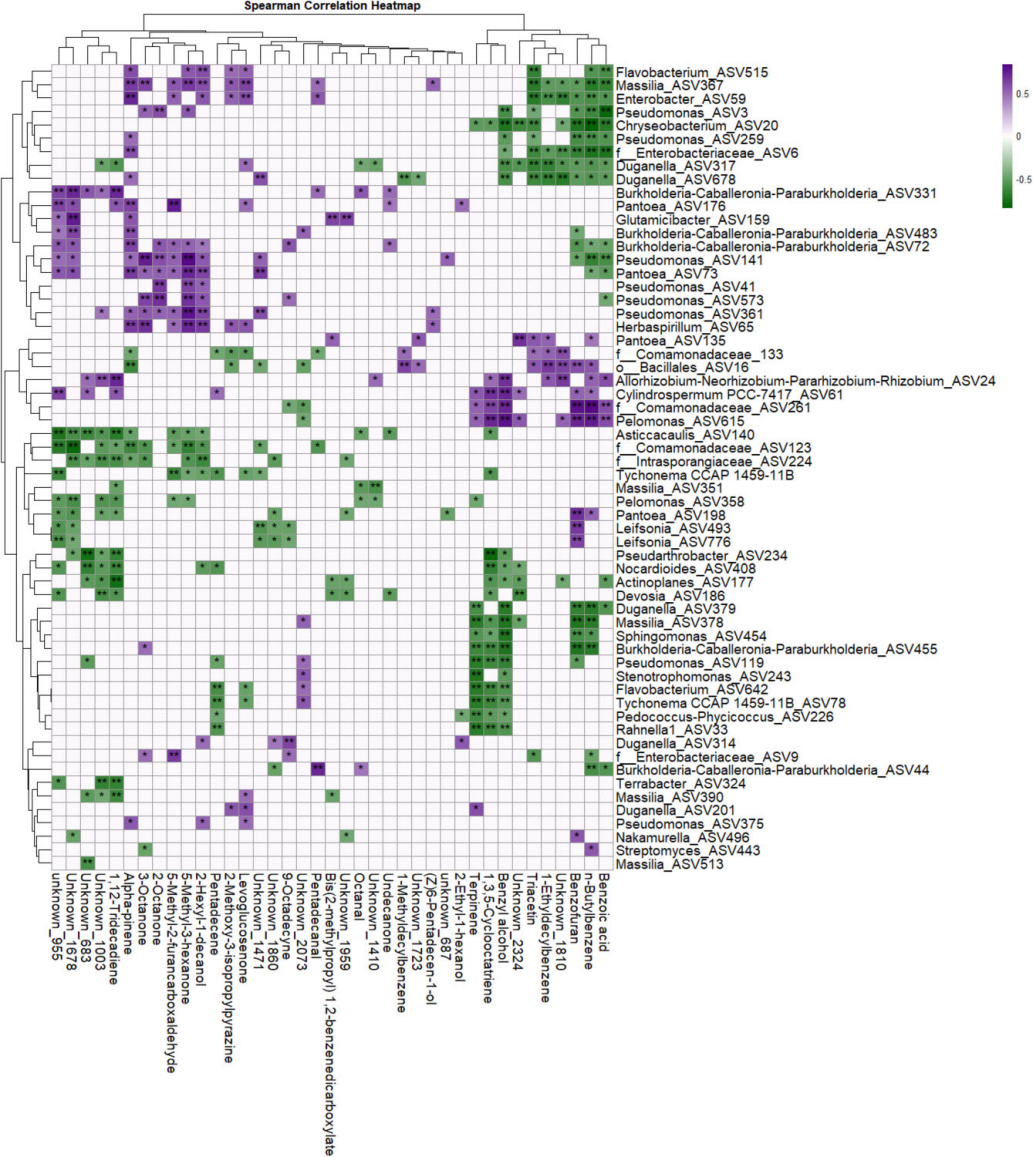
Spearman’s correlations between differentially abundant rhizoplane bacterial ASVs and root/rhizosphere-associated volatilome. Asterisks indicate significant correlations (FDR < .05) and (FDR < .01) marked with (*) and (**), respectively. Significantly positive correlations are purple colored whereas significantly negative correlations are green colored. No significant (*P* > .05) correlations was left uncolored/blank.

**Table 5.** Mantel test using Spearman’s correlation method with 999 permutations to assess the correlation between root-associated volatilome and bacterial and fungal communities in the rhizosphere and rhizoplane.

Correlation	Mantel R	*P* value
Volatile*rhizosphere bacteria	0.05	.265
Volatile*rhizoplane bacteria	0.27	.002*
Volatile*rhizosphere fungi	0.108	.123
Volatile*rhizoplane fungi	−0.037	.609

* Significant correlation (*P* < .05).

## Discussion

Our study revealed that for both the wild and domesticated tomato genotypes, foliar infection by *Botrytis* led to compositional changes in the root-associated microbiota. These compositional changes in bacterial communities were more distinct for the rhizoplane than for the rhizosphere. This compartment-specific differentiation in microbiome response is consistent with results of earlier studies on tomato as well as *Arabidopsis* and rice plants (Bulgarelli et al. 2012, Edwards et al. 2015, French et al. 2020). Overall, the root compartment poses selective pressure on the composition of microbial communities creating niches with specific microbial taxa. Such niche specificity can be explained by the multiple selection processes imposed by the plants, and/or by the ability of soil microorganisms to occupy only certain rhizocompartments (Liu et al. 2024). For the fungal community, however, no compartment-specific changes were observed, consistent with earlier studies on other plant species such as *Broussonetia papyrifera* and *Lycium ruthenicium* (Chen et al. 2020, Li et al. 2022). Collectively, these results confirm and extend earlier observations of differential responses between the two microbial domains across compartments in the soil–root interface.

Proteobacteria [currently known as *Pseudomonadota*] gradually increasingly became a dominant bacterial phylum as it moved closely to the root surface, with its highest abundance in rhizoplane. Previous studies on tomato plants showed a similar trend, with the higher abundance of this phyla in the rhizosphere and rhizoplane relative to bulk soil (Lee et al. 2019, French et al. 2020). Our result also showed higher abundance of Cyanobacteria in the tomato rhizoplane. Although their presence across rhizocompartments generally has received less attention, this phyla was reported to actively colonize the rhizoplane of rice and hairgrass species *Deschampsia antartica*, which may be associated with their competitive ability to form biofilms on root surfaces (Ahmed et al. 2010, 2014, Znój et al. 2022). Different concentrations of root metabolites on root surface than the adjacent soil layers may give additional selection pressure on the rhizoplane-inhabiting microbes due to distinct capability of the individual taxa to metabolize or tolerate certain compounds. Also differences in root architecture between the plant genotypes (not studied in this research) can shape rhizoplane microbial communities (Herms et al. 2022). A previous study on wild and domesticated common beans revealed that root phenotypic traits (e.g. specific root length) contributed to 11.4% of the variation in rhizobacterial community composition (Pérez-Jaramillo et al. 2017).

Foliar stress caused by *Botrytis* generally induced bacterial ASV changes in a genotype-specific manner. *Botrytis* infection reduced the abundance of Proteobacteria in the rhizoplane of *S. pimpinellifolium*, which was exemplified by the lower abundances of ASVs classified as *Pseudomonas, Duganella, Devosia*, and *Massilia*. Strains from these genera have been commonly described for their plant growth promotion and disease-suppressive effects in various plant species (Yin et al. 2013, 2021). For example, the genus *Massilia* was previously found to be enriched in healthy *S. pimpinellifolium* across several growth cycles, suggesting that such genera might be actively recruited by this wild tomato genotype under optimal growth conditions (Cordovez et al. 2021). *Botrytis* infection of *S. lycopersicum* var. Moneymaker caused depletion of several ASVs classified as *Flavobacterium* and *Sphingomonas*. The genus *Sphingomonas* has been previously reported to promote tomato growth and induce tomato resistance to oxidative stress under salinity (Khan et al. 2014, Halo et al. 2015), while *Flavobacterium* can suppress bacterial wilt incident caused by *Ralstonia solanacearum* in *S. lycopersicum* var. Moneymaker (Kwak et al. 2018). Apart from the taxa depletion, we also observed a higher relative abundance of several taxa in the rhizoplane of two tomato genotypes infected by *B. cinerea*, in particular ASVs classified as *Pelomonas, Cylindrospermum, Streptomyces*, and unknown genera belonging to the family *Comamonadaceae. Streptomyces* strains are known to induce systemic resistance of chickpea plants against *B. cinerea* and of tomato plants against leaf curl virus (Vijayabharathi et al. 2018, Chen et al. 2022). Interestingly, *Cylindrospermum* has been recently reported to be a key rhizosphere taxon responsible for systemic resistance in *S. lycopersicum* (Ketehouli et al. 2024). Furthermore, the rhizosphere-enriched *Comamonadacae* family has been reported to induce resistance against *Fusarium oxysporum* in cucumber plants (Ketehouli et al. 2024).

Taxa depletion was also found for fungal ASVs, mainly from Ascomycota and Mortierellomycota, with a reduced relative abundance of *Mortierella* and *Chaetomium* in the rhizosphere and rhizoplane of *Botrytis*-infected *S. pimpinellifolium. Mortierella* was previously described as a plant growth-promoting fungus and biocontrol agent against *Verticillium dahliae* in tomato plants (Ozimek and Hanaka 2021, Nouri et al. 2024). This genus was also enriched in the rhizosphere of maize and was responsible for soil carbon and phosphorous cycling. Strains from the genus *Chaetomium* are often referred to as biocontrol agents for *Fusarium* wilt in tomato plants due to their ability to produce antimicrobial compounds and to induce systemic resistance (Madbouly et al. 2017, Singh et al. 2021). Infection of *S. lycopersicum* var. Moneymaker by *Botrytis*, on the other hand, led to a reduced abundance of ASVs classified as *Fusarium* and *Lachnum. Fusarium* genera are commonly regarded as soil-borne pathogens of different plant species and their presence can negatively impact on crop growth and productivity. The genus *Lachnum* was previously studied for its colonization and plant growth-promoting effects on blueberry plants (Bizabani and Dames 2015). Interestingly, we also found enrichment of ASVs classified as *Trichoderma* in the rhizosphere and rhizoplane of both tomato genotypes. In line with our finding, a previous study demonstrated the enrichment of *Trichoderma* strain T34 in the rhizosphere of tomato plants infected with *B. cinerea*. When inoculated on tomato, this strain induced systemic resistance against the same foliar pathogen (Fernández et al. 2017), exemplifying that upon infection the plant may recruit or enrich microbiome members that trigger a resistance response for protection against subsequent infections. To what extent this combination of taxa depletion and enrichment affects plant growth and stress resistance remains to be investigated.

Recently, we showed that the profile of root volatile organic compounds (rVOCs) differed between *S. pimpinellifolium* and *S. lycopersicum* var. Moneymaker, with higher abundance of monoterpene compounds detected in the wild tomato (Díaz et al. 2022). Our *in vitro* study further confirmed that roots of *S. pimpinellifolium* emit higher monoterpene compounds such as α-pinene, camphene, limonene, and terpinene. Furthermore, our previous study also showed that aboveground insect herbivory altered the rhizosphere volatilome of tomato (Lee Díaz et al. 2024). In the present study, we also showed that *Botrytis* leaf infection changed the tomato rhizosphere volatilome quantitatively as well as qualitatively. These changes were largely unique to each of the two tomato genotypes tested. For instance, a higher intensity (based on peak areas) of 1,2-tridecadiene was detected in the rhizosphere of infected *S. pimpinellifolium*, while a lower intensity of 5-methyl-3-hexanone was detected in the same treatment. Volatile analyses of infected *S. lycopersicum* var. Moneymaker showed an increased intensity of terpinene. Terpinene is a compound classified as monoterpene and is known to be emitted by several plant species including tomato and pine (Lin et al. 2007, Díaz et al. 2022). This compound was also emitted in response to phytohormone stress and exhibits antimicrobial properties, which in turn may impact microbial community structure (Alavi-Samani et al. 2015, Yang et al. 2022).

The Mantel test results indicate a significant correlation between the rhizosphere-associated volatilome and the rhizoplane rather rhizosphere bacterial community (*r* = 0.27, *P* = .002), while no such correlation was found for fungal communities in either the rhizoplane or rhizosphere. This suggests different responsiveness among microbial communities toward certain root metabolites. Higher exudate concentrations at the root surface compared to the adjacent soil layer may act as a stronger driver of bacterial community assembly, leading to the differing responsiveness of rhizoplane and rhizosphere bacteria to volatile compounds. However, while our study focused on volatile compounds, it is important to recognize that roots also release nonvolatile, water-soluble compounds that can play crucial roles in shaping microbial assembly (Zhalnina et al. 2018, Koprivova and Kopriva 2022). Therefore, rVOCs should be viewed as one of several drivers influencing rhizoplane community structure, rather than the sole determinant.

Whether the volatiles measured in this study are produced directly by plant roots or rhizoplane microbes requires further scrutiny. It is also plausible that both plants and microbes emit specific volatiles in response to their dynamic interactions, further complicating the source attribution of these compounds. Interestingly, benzyl alcohol and benzofuran were detected in the rhizosphere of both tomato genotypes upon foliar stress. These two compounds were also detected in our *in vitro* experiment, emitted by roots of both tomato genotypes. Previously, benzyl alcohol and benzofuran were also detected in the root of tomato plants upon herbivory stresses (Díaz et al. 2022, Lee Díaz et al. 2024), suggesting that these compounds may be more generic markers of root volatiles of plants under biotic stresses.

Spearman’s correlation analyses showed positive associations between stress-associated volatiles such as benzyl alcohol, benzofuran, and *n*-butyl benzene to bacterial taxa enriched in rhizoplane of both tomato genotypes such as *Pelomonas, Cylindrospermum*, and *Comamonadaceae*, while taxa such as *Enterobacteriaceae* and *Chryseobacterium* correlated negatively. These findings suggest root-associated volatiles might, in part, selectively shape bacterial populations, potentially influencing microbial dynamics and plant defense mechanisms (Schulz-Bohm et al. 2018, de la Porte et al. 2020, Rizaludin et al. 2021).

Overall, our results show that tomato plants systemically respond to foliar infection by altering the composition of root-associated microbial communities and the rhizosphere/root volatilome. Future research needs to be performed to understand the reciprocal effects of these microbial and volatile changes for plant growth and stress resilience.

## Supplementary Material

fiaf042_Supplemental_Files

## Data Availability

Raw sequences data (both 16S and ITS amplicons) are available at SRA NCBI under BioProject number PRJNA1241739. Raw GC-MS data are publicly available at Zenodo: 10.5281/zenodo.14223861. The code used to analyzed both 16S and ITS amplicon data is available at Github: https://github.com/MuhammadRizaludin/Botrytis.
