# Supplementary material for: Foliar infections by Botrytis cinerea modulate the tomato root volatilome and microbiome
Source: FEMS Microbiol Ecol. 2025 Apr 18;101(5):fiaf042. doi: 10.1093/femsec/fiaf042 (PMC12023855; doi:10.1093/femsec/fiaf042)
Supplement: fiaf042_Supplemental_Files [file fiaf042_supplemental_files.zip › Description of Supplementary Data.docx]

**Description of Supplementary Data**

**Supplementary Data 1**

**Description:** List of mass features detected in all treatments (sheet 1), and those that are significantly enriched (compared to unplanted bulk soil) in rhizosphere of uninfected (healthy)

*S.pimpinellifolium* (sheet 2), in infected (stressed*) S. pimpinellifolium* (sheet 3), in uninfected

(healthy) *S.lycopersicum* (sheet 4) and in infected (stressed) *S.lycopersicum* and the combined

mass features that were significantly enriched in the pairwise comparisons with unplanted bulk

soil (therefore called rhizosphere/root-associated volatilome).

**Supplementary Data 2**

**Description:** All pairwise comparison results of mass features detected across treatments: healthy

*S.pimpinellifolium* vs healthy *S.lycopersicum* (sheet 1), healthy vs stressed *S.pimpinellifolium*

(sheet 2), healthy vs stressed *S.lycopersicum* (sheet 3), stressed *S.pimpinellifolium* vs stressed

*S.pimpinellifolium* (sheet 4), healthy *S.pimpinellifolium* vs stressed *S.lycopersicum* (sheet 5),

healthy *S.lycopersicum* vs stressed *S.pimpinellifolium* (sheet 6), and combined unique features of

all significant pairwise comparisons and their identities (sheet 7)

**Supplementary Data 3**

**Description:** All pairwise comparison (Deseq2 contrast) results of rhizoplane bacterial ASVs

detected across treatments: healthy vs stressed *S.pimpinellifolium* (sheet 1), healthy vs stressed

*S.lycopersicum* (sheet 2), healthy *S.pimpinellifolium* vs healthy *S.lycopersicum* (sheet 3), and

stressed *S.pimpinellifolium* vs stressed *S.pimpinellifolium* (sheet 4), healthy *S.pimpinellifolium* vs

stressed *S.lycopersicum* (sheet 5), healthy *S.lycopersicum* vs stressed *S.pimpinellifolium* (sheet 6)

**Supplementary Data 4**

**Description:** All pairwise comparison (Deseq2 contrast) results of rhizosphere fungal ASVs

detected across treatments: healthy vs stressed *S.pimpinellifolium* (sheet 1), healthy vs stressed

*S.lycopersicum* (sheet 2), healthy *S.pimpinellifolium* vs healthy *S.lycopersicum* (sheet 3), and

stressed *S.pimpinellifolium* vs stressed *S.pimpinellifolium* (sheet 4), healthy *S.pimpinellifolium* vs

stressed *S.lycopersicum* (sheet 5), healthy *S.lycopersicum* vs stressed *S.pimpinellifolium* (sheet 6)

**Supplementary Data 5**

**Description:** All pairwise comparison (Deseq2 contrast) results of rhizoplane fungal ASVs

detected across treatments: healthy vs stressed *S.pimpinellifolium* (sheet 1), healthy vs stressed

*S.lycopersicum* (sheet 2), healthy *S.pimpinellifolium* vs healthy *S.lycopersicum* (sheet 3), and

stressed *S.pimpinellifolium* vs stressed *S.pimpinellifolium* (sheet 4), healthy *S.pimpinellifolium* vs

stressed *S.lycopersicum* (sheet 5), healthy *S.lycopersicum* vs stressed *S.pimpinellifolium* (sheet 6)
