# Supplementary material for: Foliar infections by Botrytis cinerea modulate the tomato root volatilome and microbiome
Source: FEMS Microbiol Ecol. 2025 Apr 18;101(5):fiaf042. doi: 10.1093/femsec/fiaf042 (PMC12023855; doi:10.1093/femsec/fiaf042)
Supplement: fiaf042_Supplemental_Files [file fiaf042_supplemental_files.zip › revised_Supplementary Figures.docx]

**Foliar infections by *Botrytis cinerea* modulate the tomato root volatilome and microbiome**

Muhammad Syamsu Rizaludin^1*^, Ana Shein Lee Diaz^1^, Hans Zweers^1^, Jos M. Raaijmakers^1,2^ and Paolina Garbeva^1*^

1. The Netherlands Institute of Ecology (NIOO-KNAW), Department of Microbial Ecology, Droevendaalsesteeg 10, 6708 PB, Wageningen, The Netherlands
2. Leiden University, Institute of Biology, Slyviusweg 2333 BE, Leiden, The Netherlands

*Correspondence: [M.Rizaludin@nioo.knaw.nl](mailto:M.Rizaludin@nioo.knaw.nl), [p.garbeva@nioo.knaw.nl](mailto:p.garbeva@nioo.knaw.nl)

**Supplementary Figures**


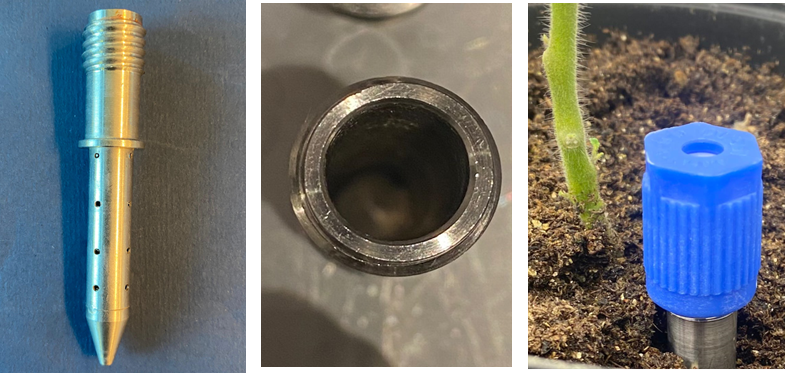


**Supplementary Figure S1**. Non-invasive set-up to sample root/rhizosphere associated volatilome in planta. Hisorb probes were inserted into cylindrical holders made of stainless steels, and buried for approximately 5 cm deep in the soil near tomato roots to trap root-associated volatiles. The cylindrical holders have small perforated surface preventing soil contact, creating headspace and allowed passive diffusion of volatile compounds toward the probes inside the holders. Screw caps on the holders enabled secure closure to minimize contamination of volatile compounds emitted by aboveground plant tissue or by environmental backgrounds.


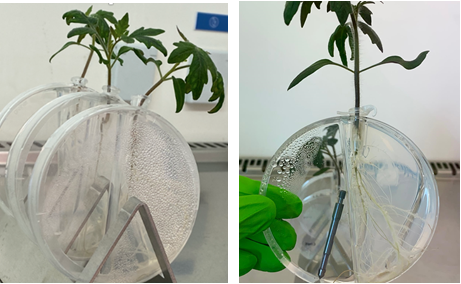

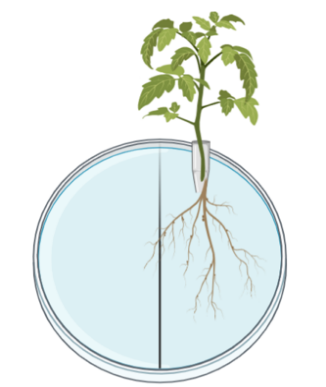

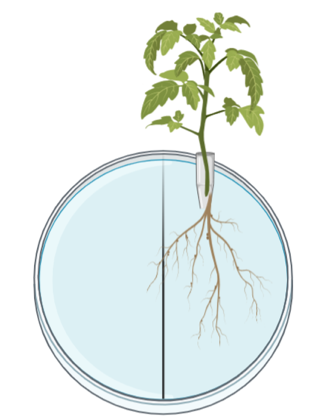


Healthy

*Botrytis-infected*


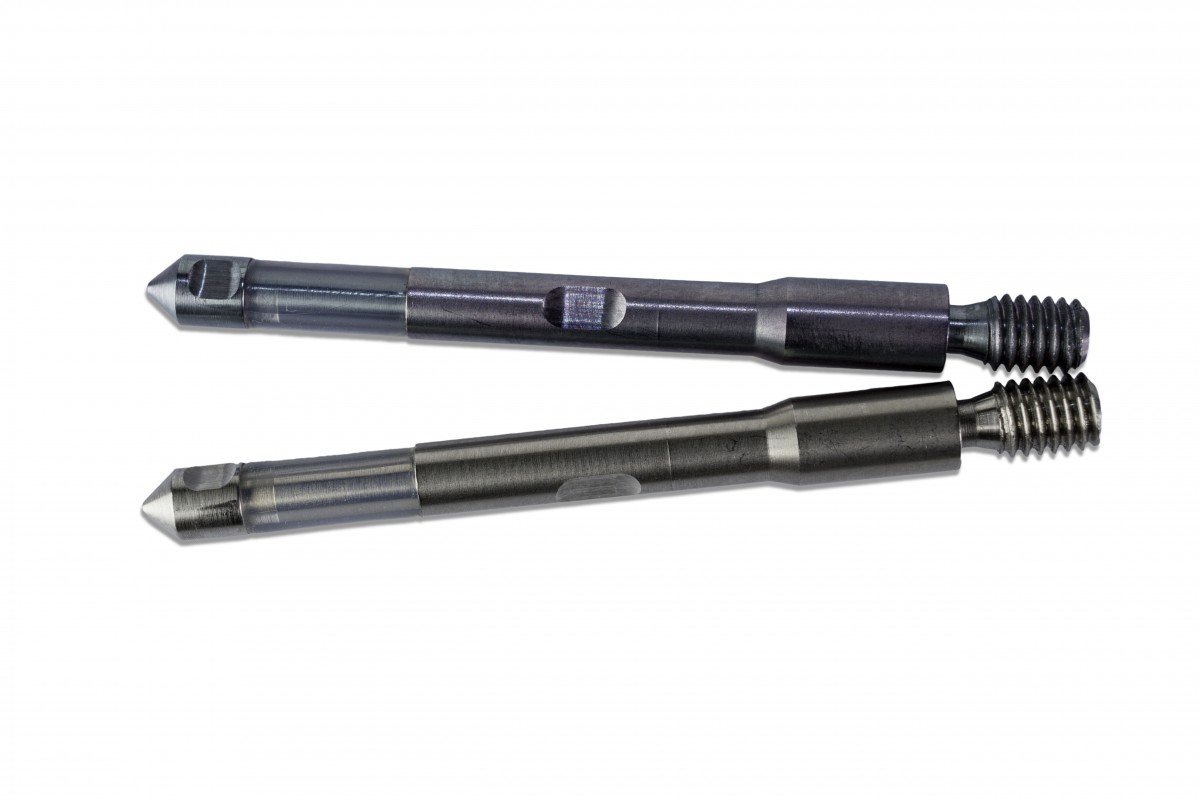

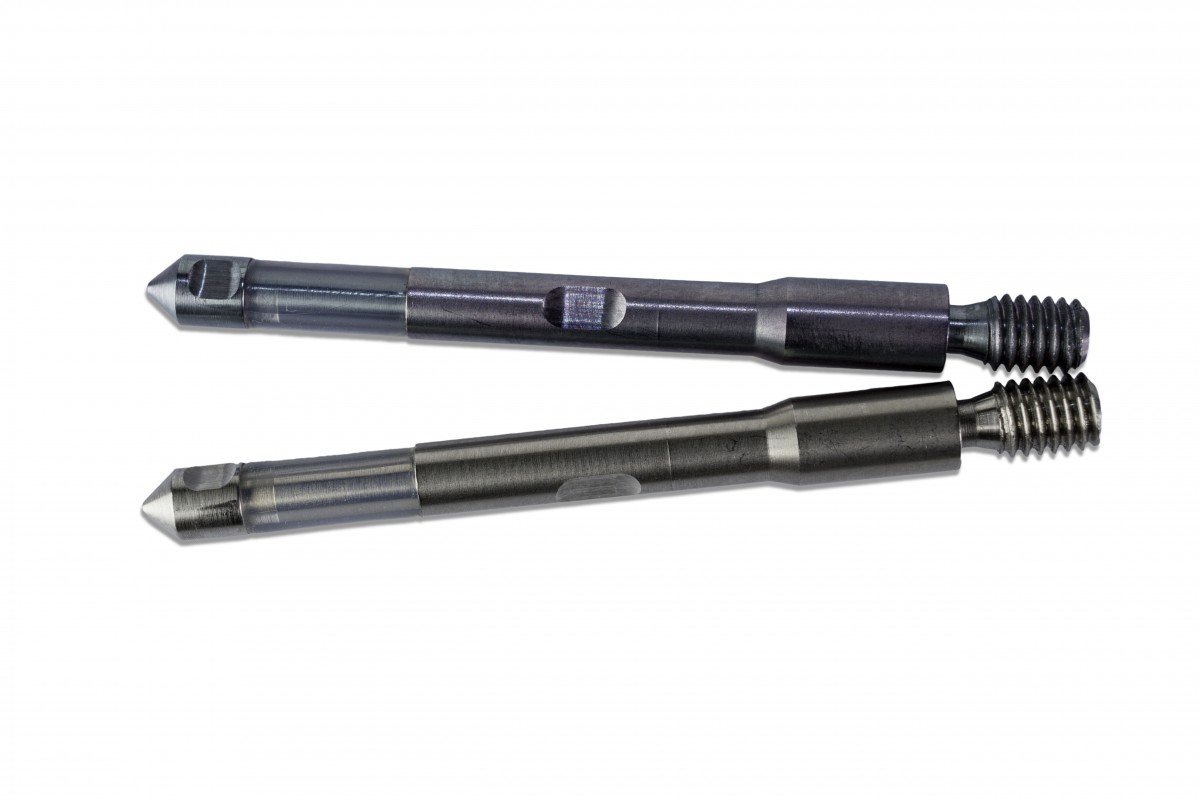

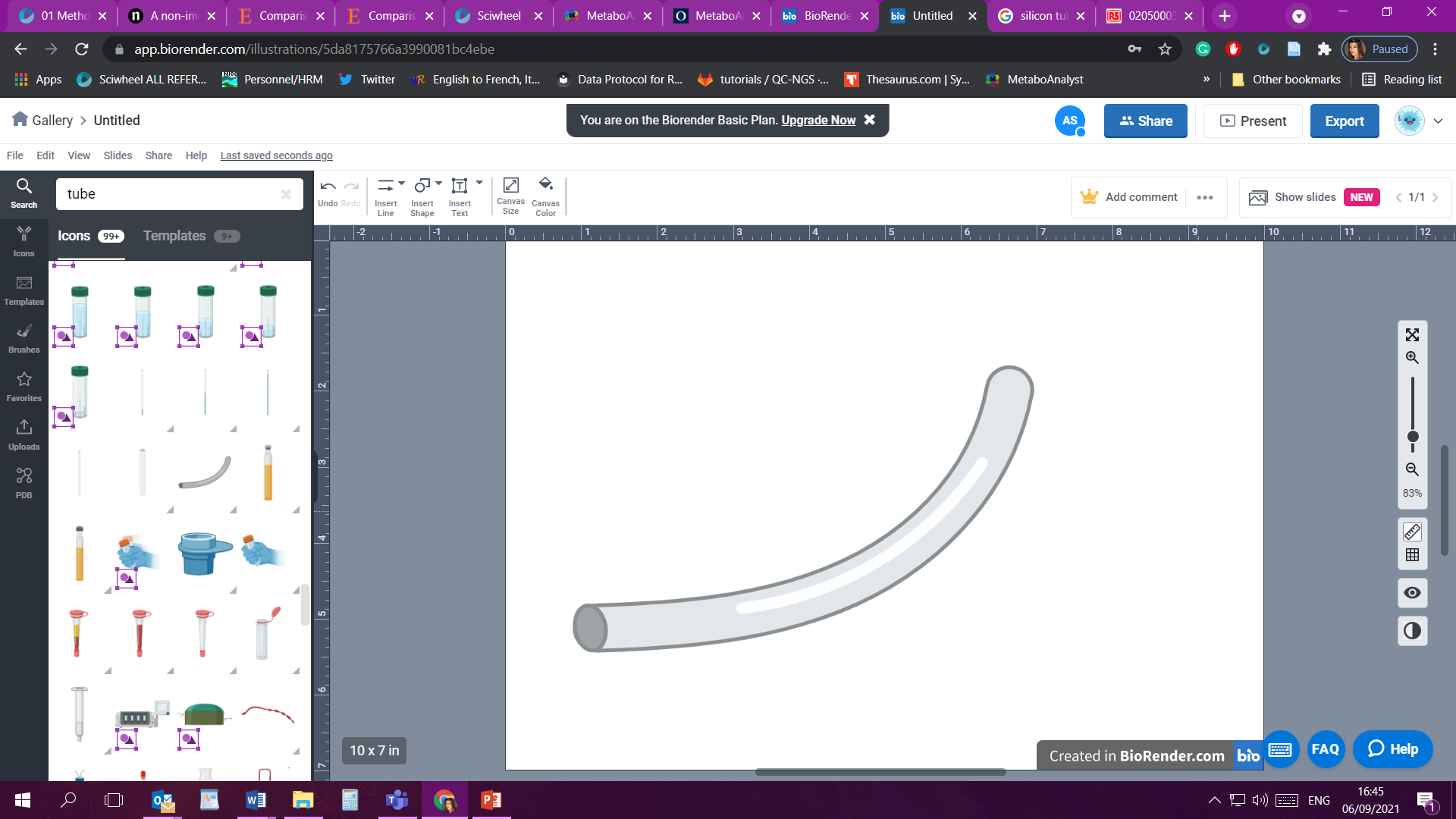

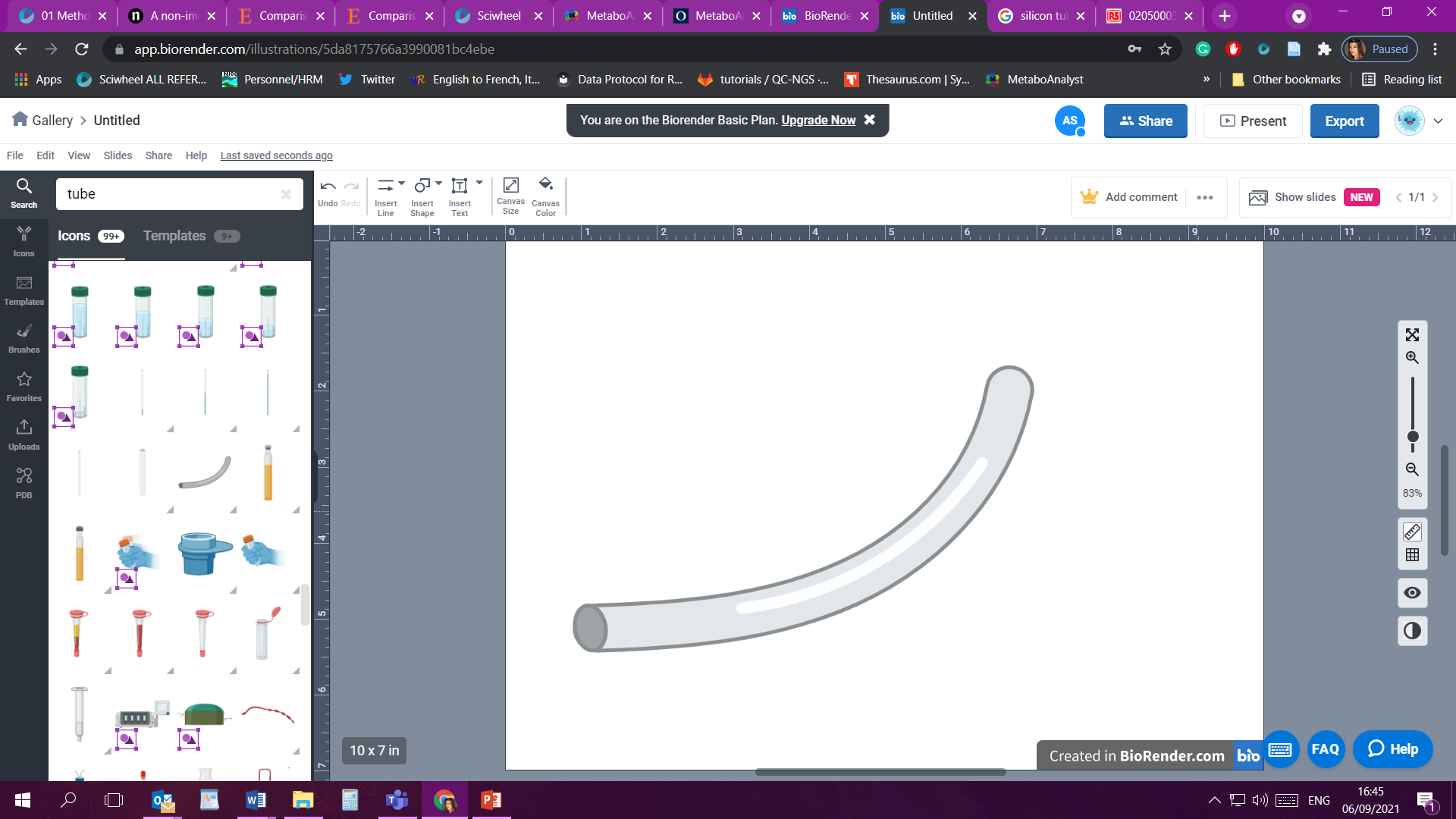

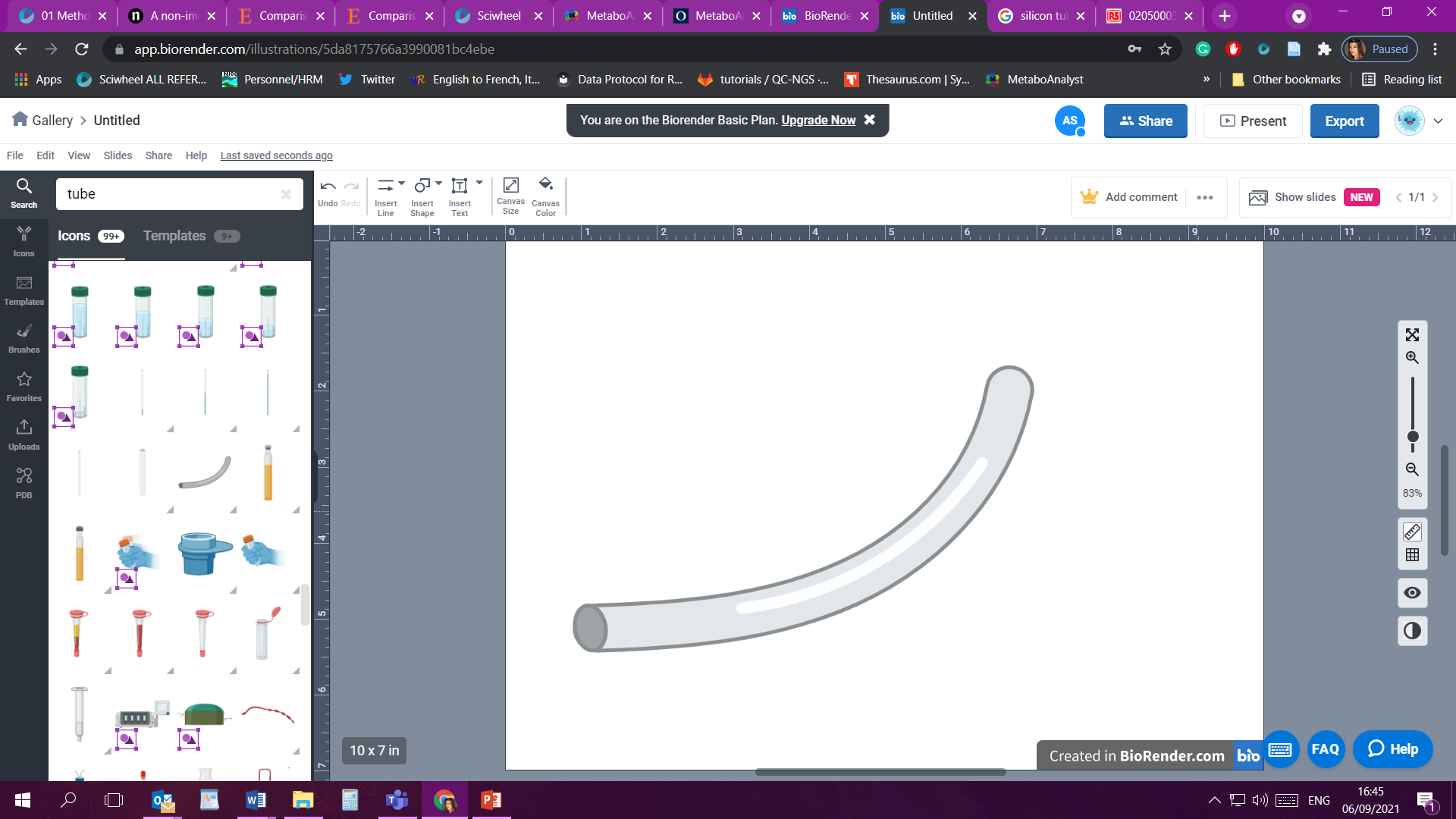

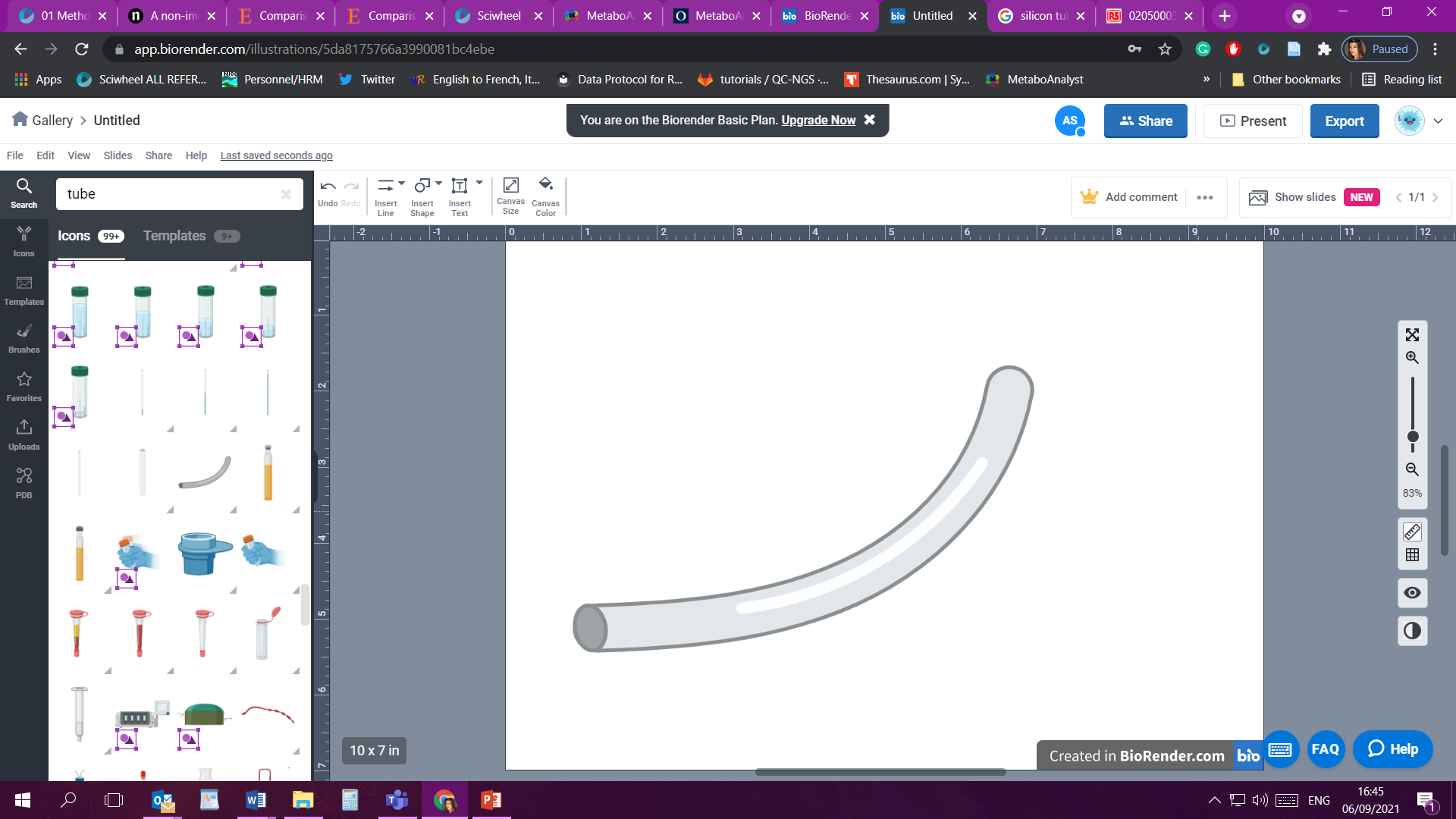


**Supplementary Figure S2.** Low-disturbance sampling of root volatile compounds in an in-vitro system. Tomato plants were grown (under sterile condition) using Murashige and Skoog (MS) medium filled at one side of the two-compartment petri plate. The roots grew along the MS media inside the petri plate, while tomato shoots grew outside the petri plate. Another side of the compartment remained empty (headspace) for placing the sorbent material (HiSOrb). The two-compartment plate enabled volatile compounds emitted by roots (in one compartment) to passively diffuse toward the headspace containing HiSorb probe allowing the collection of root volatile compounds. 48h following VOC collection, tomato leaves were (un)infected with B.cinerea.

**A)**

**B)**

***S.lycopersicum***

***S.pimpinellifolium***


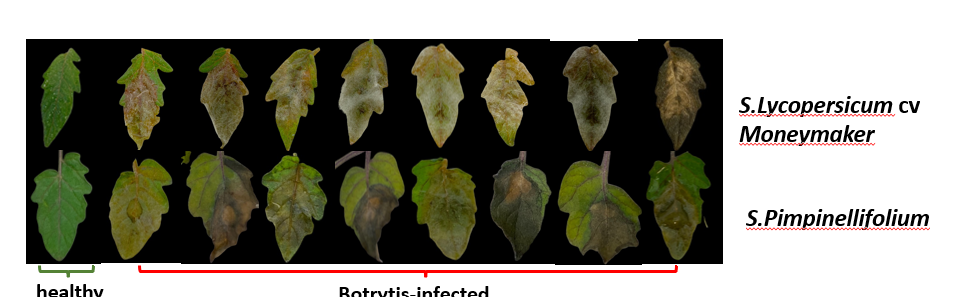

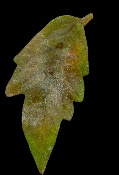

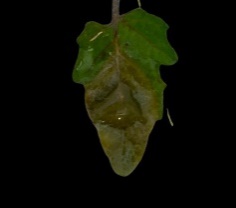

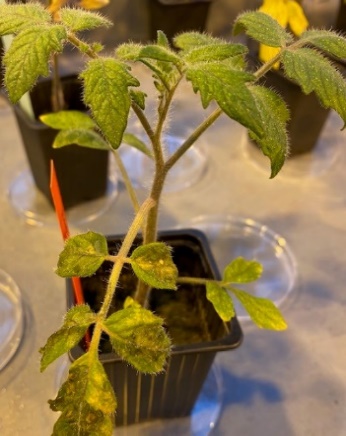

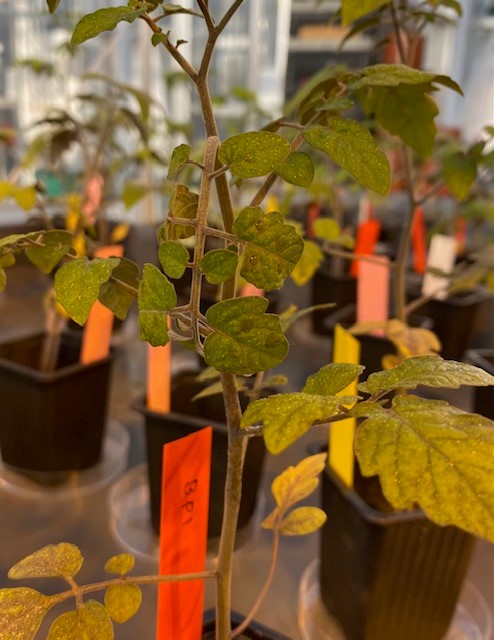


***S.pimpinellifolium***

***S.lycopersicum***

*****


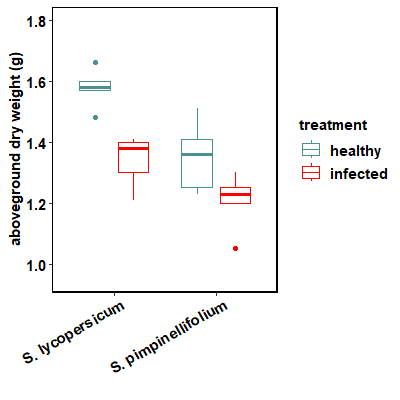


***p*=0.04**

***p*=0.001**


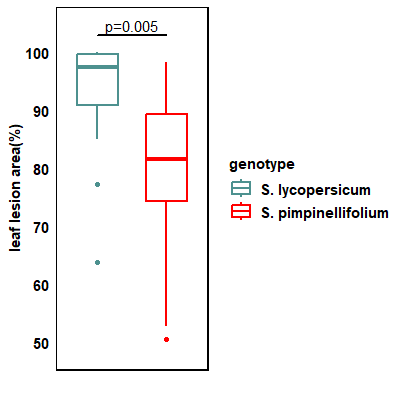


**Supplementary Figure S3.** Impact of the foliar infection by Botrytis cinerea on the disease progression and aboveground biomass in a wild tomato S. pimpinellifolium and a domesticated tomato S. lycopersicum. **(A)** The y-axis shows lesion percentage (%) indicating the progression of disease in the leaflet of the wild and the modern tomatoes after four days post-inoculation with spore suspension of Botrytis cinerea in a detached leaf bioassay. WINFOLIA^TM^ software was utilized to determine the lesion area (discoloured tissue), to quantify the percentage (%) of infected over the non-infected leaflet areas. **(B)** The dry shoot biomass of healthy (n=5 independent biological replicates) and non -infected tomato plants (n=5 independent biological replicates) after two weeks of the fungal infection . The individual point in the bar plot refer to a single replicate in a treatment (A,B). Statistical difference values (p) were obtained following the t.test.

**(A)**


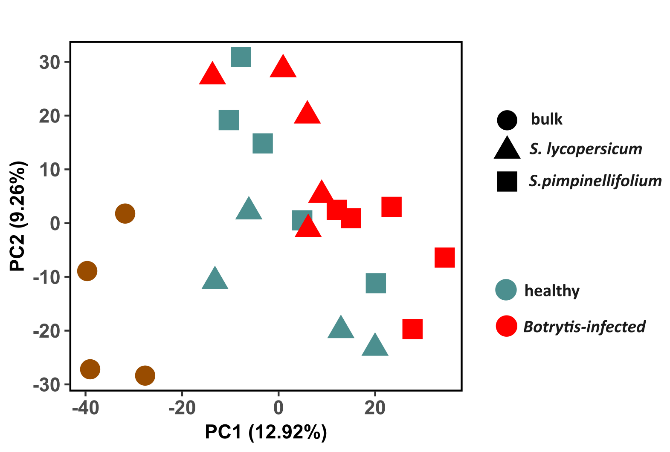

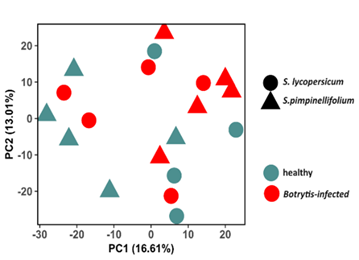


**(B)**

**Supplementary Figure S4. (A)** The principal coordinate analysis (PCoA) representing the dissimilarity (based on the Aitchison distance ) of total volatilome (unsubstracted) originated from bulk (circle, brown colored) and from root/rhizosphere of S.lycopersicum cv. Moneymaker (triangle) and S.pimpinellifolium (triangle), with each genotype under infection with B.cinerea (Botrytis-infected, red colored) and without infection ( healthy, green colored). **(B)** The principal coordinate analysis (PCoA) representing the dissimilarity (based on the Aitchison distance ) of filtered data set (only rhizosphere-associated volatile profile) between two genotypes: S.lycopersicum cv. Moneymaker (circle) and S.pimpinellifolium (triangle), with each genotype under infection with B.cinerea (Botrytis-infected, red colored) and without infection ( healthy, green colored)

**(A)**


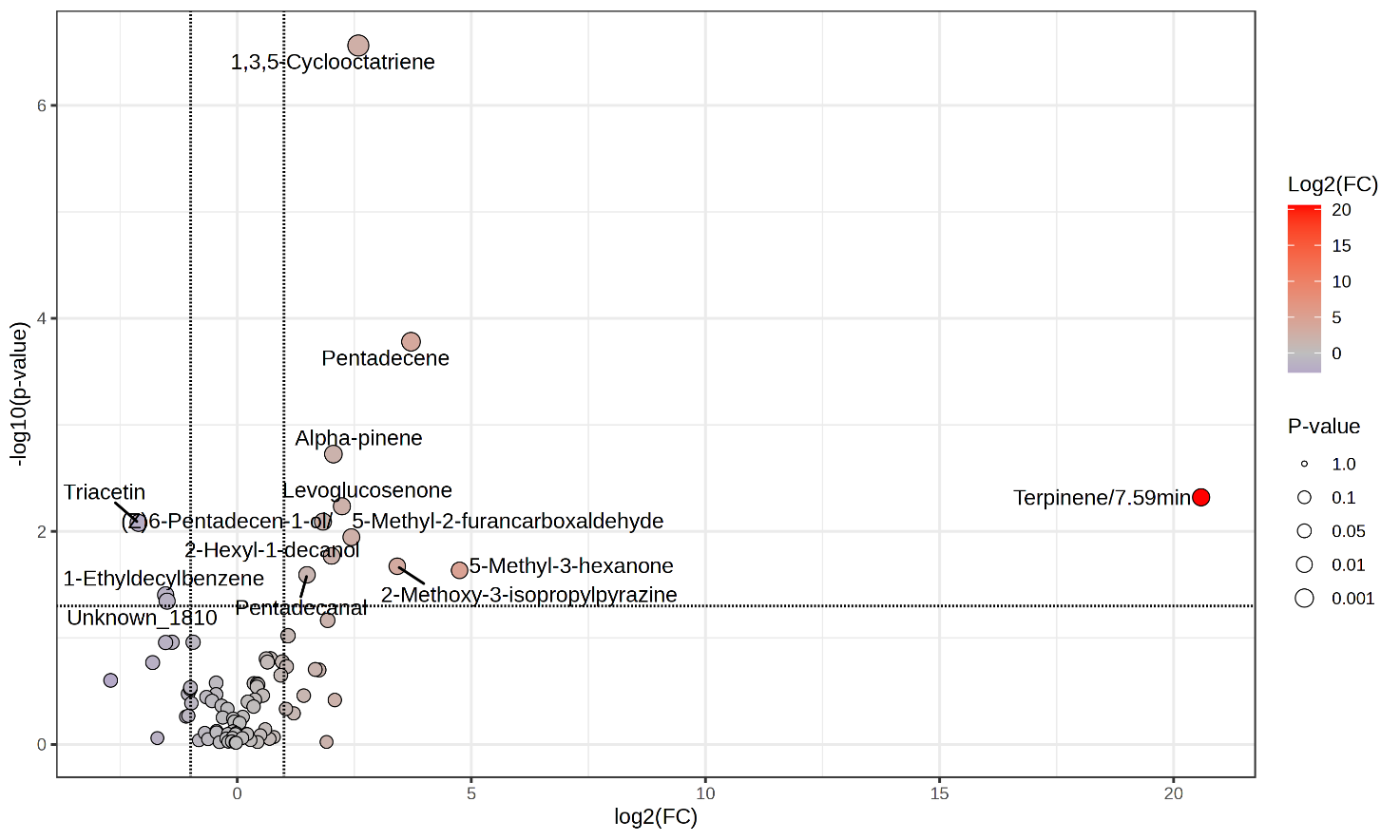


**(B)**


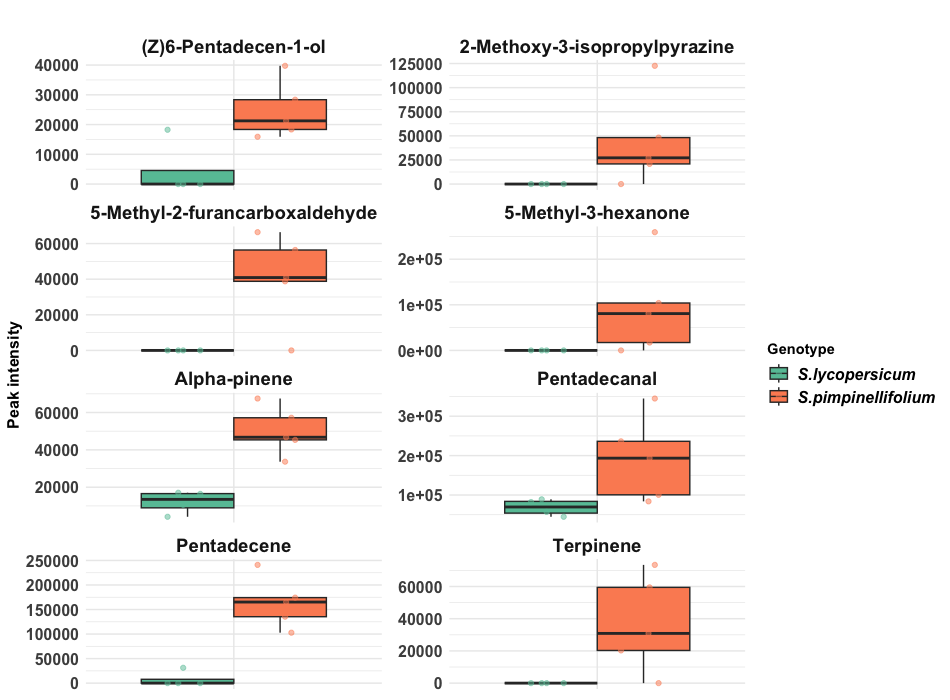


**Supplementary Figure S5.** (A) Volcano plot showing pairwise contrast VOCs enriched in S.pimpinellifolium (right side of the plot) and those that enriched in S. lycopersicum var. Moneymaker (left side of the plot) in the absence of B.cinerea infection (non-stressed condition). Alpha pinene and terpinene (monoterpenes) were among the compounds emitted at significantly higher levels in healthy S.pimpinellifolium than in S.lycopersicum var. Moneymaker. (B) Boxplots illustrating the peak intensities (peak areas) of selected VOCs that were significantly detected in the rhizosphere of S. pimpinellifolium and S. lycopersicum in the absence of Botrytis cinerea (non-infected plants). Each boxplot represents the distribution and median of peak areas from five biological replicates (n = 5) per treatment

**(A)**


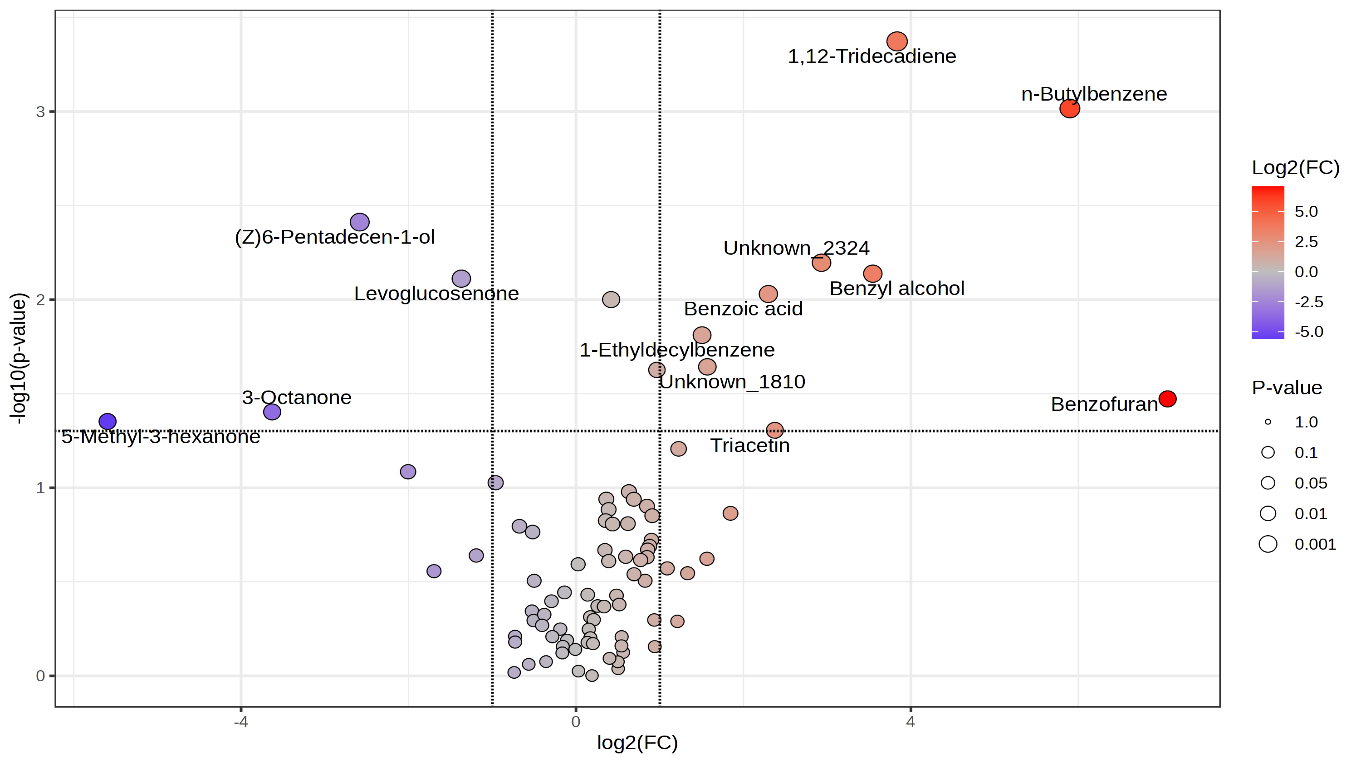


**(B)**


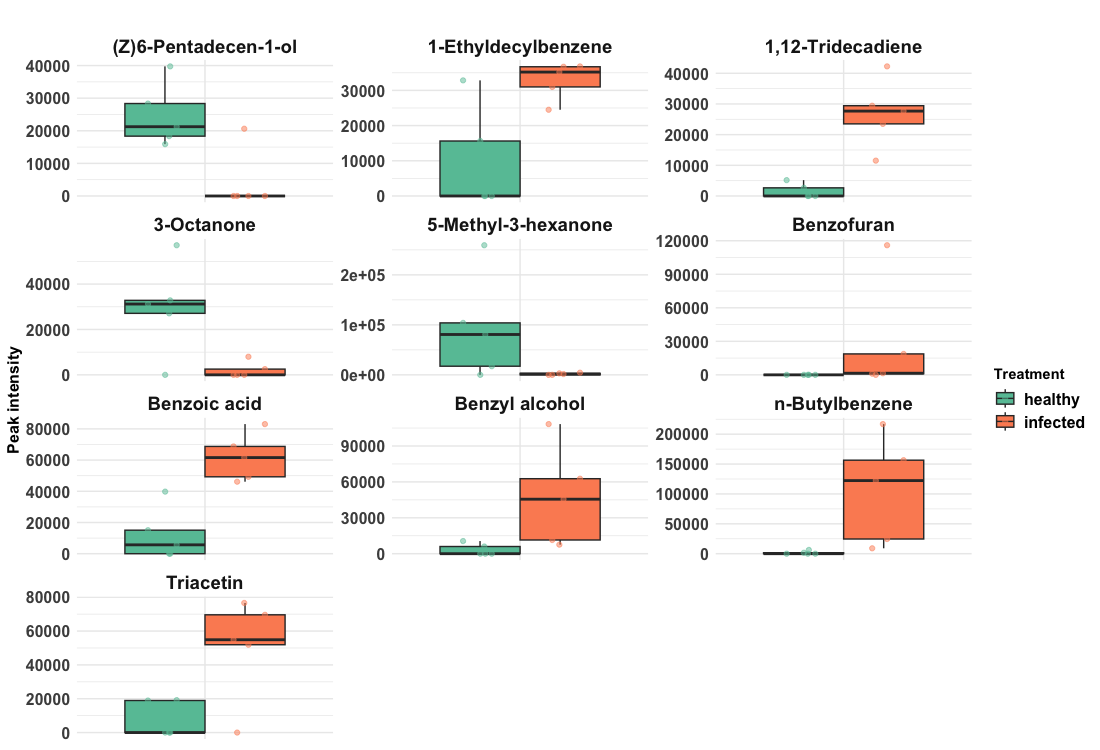


**Supplementary Figure S6.** (A) Volcano plot showing pairwise contrast of VOCs that are enriched (right side of the plot) and depleted (left side of the plot) in the rhizosphere of Botrytis-infected S.pimpinellifolium. (B) Boxplots illustrating the peak intensities (peak areas) of selected VOCs that were significantly detected in the rhizosphere of healthy and Botrytis-infected (infected) S. lycopersicum cv Moneymaker. Each boxplot represents the distribution and median of peak areas from five biological replicates (n = 5) per treatment

**
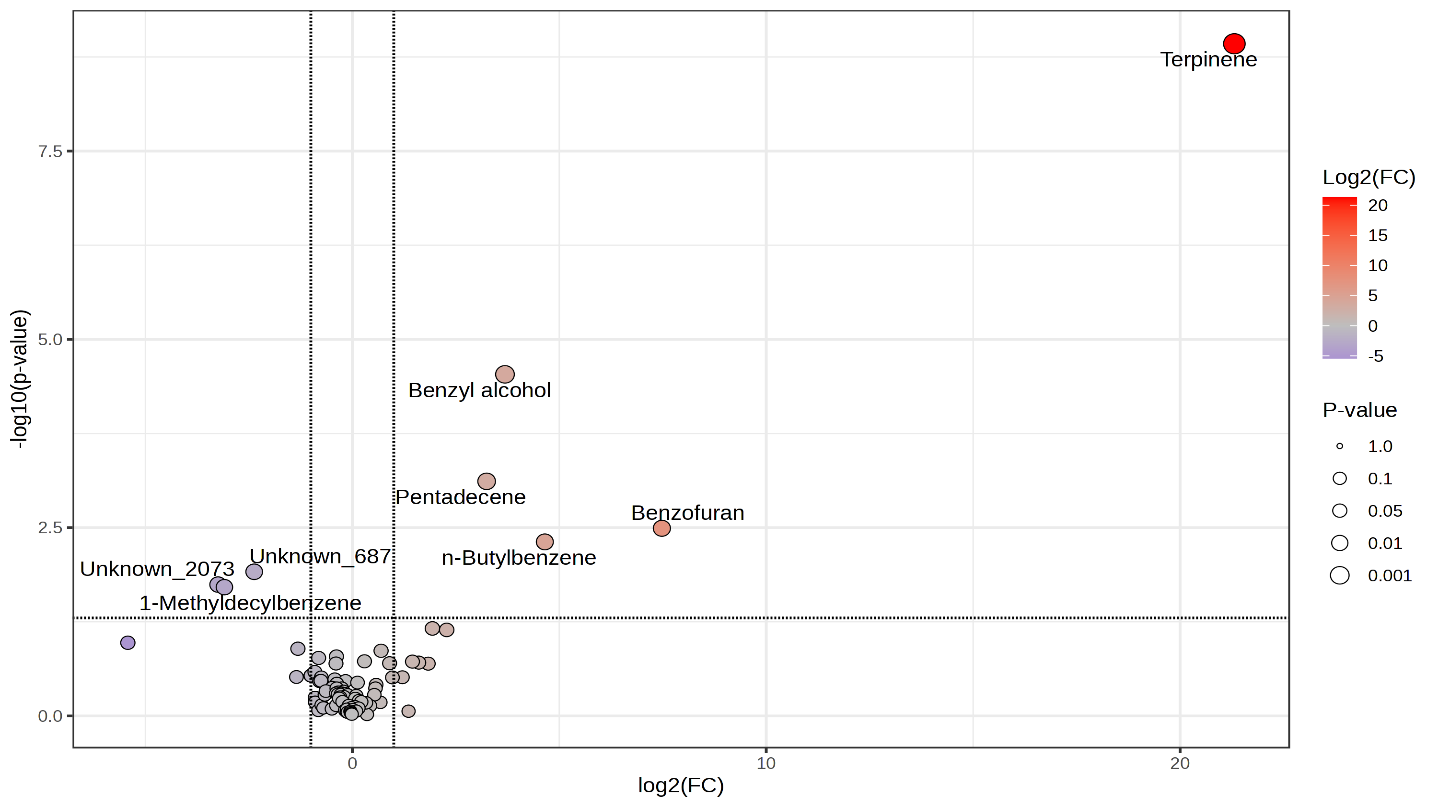
**


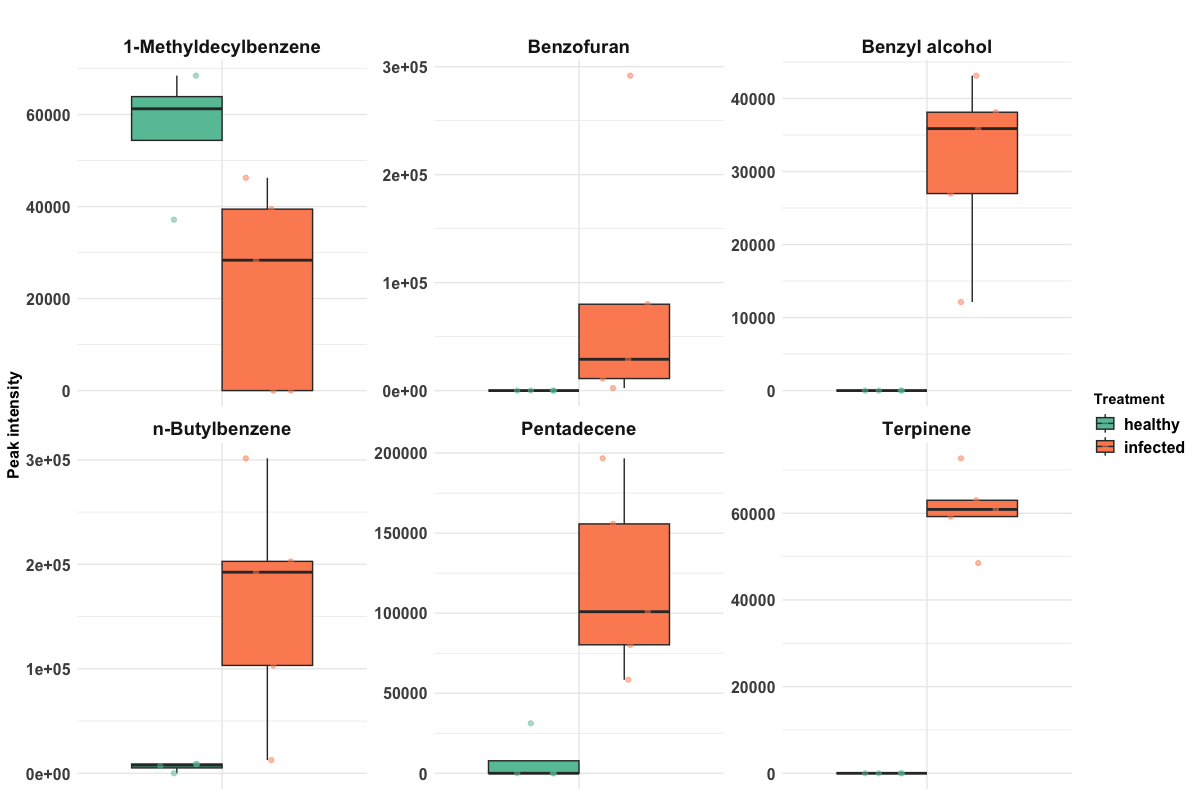


**Supplementary Figure S7.** (A) Volcano plot showing pairwise contrast of VOCs that are enriched (right side of the plot) and depleted (left side of the plot) in the rhizosphere of Botrytis-infected S.lycopersicum var. Moneymaker. (B) Boxplots illustrating the peak intensities (peak areas) of selected VOCs that were significantly detected in the rhizosphere of healthy and Botrytis-infected (infected) S. lycopersicum cv Moneymaker. Each boxplot represents the distribution and median of peak areas from five biological replicates (n = 5) per treatment

**(B)**

**(A)**


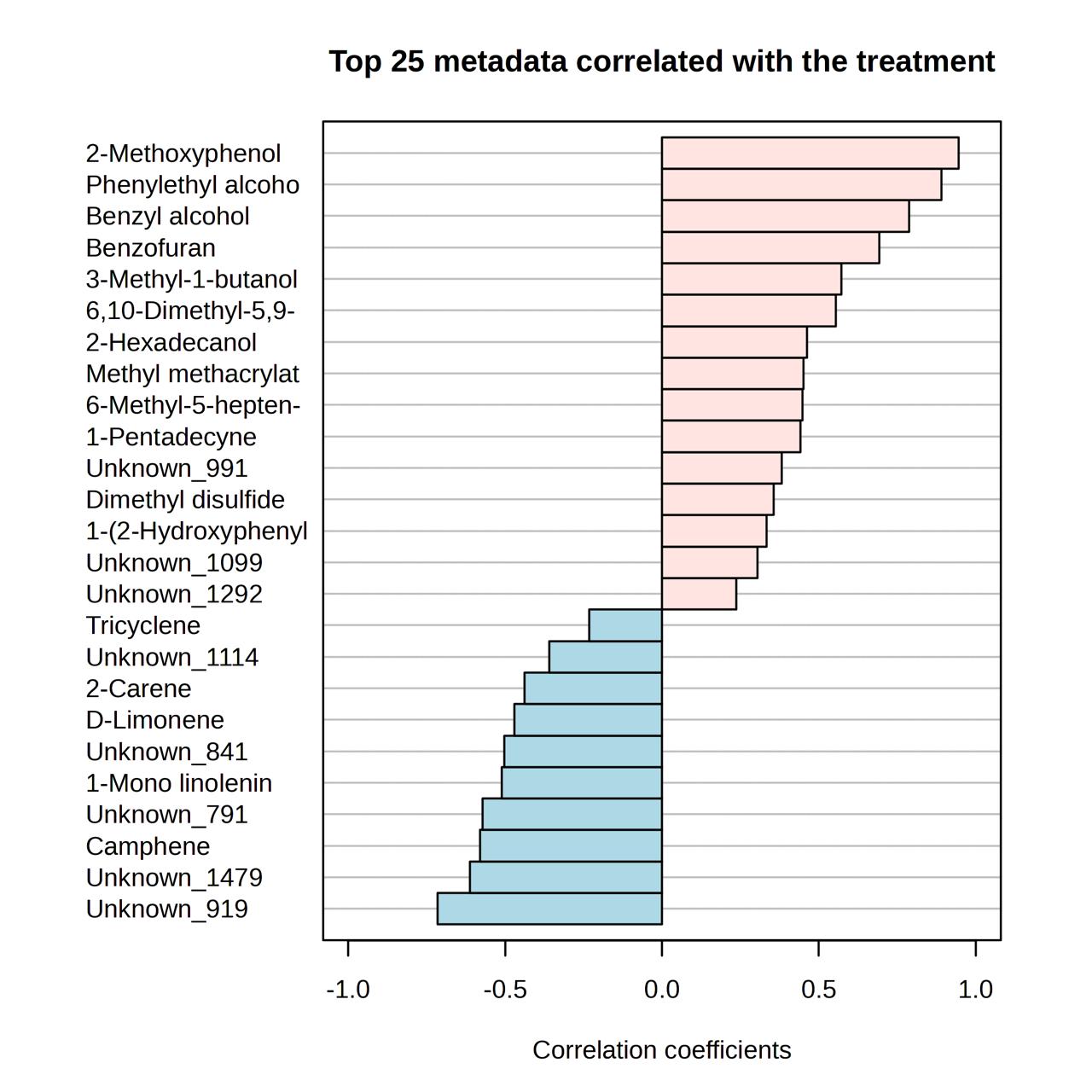

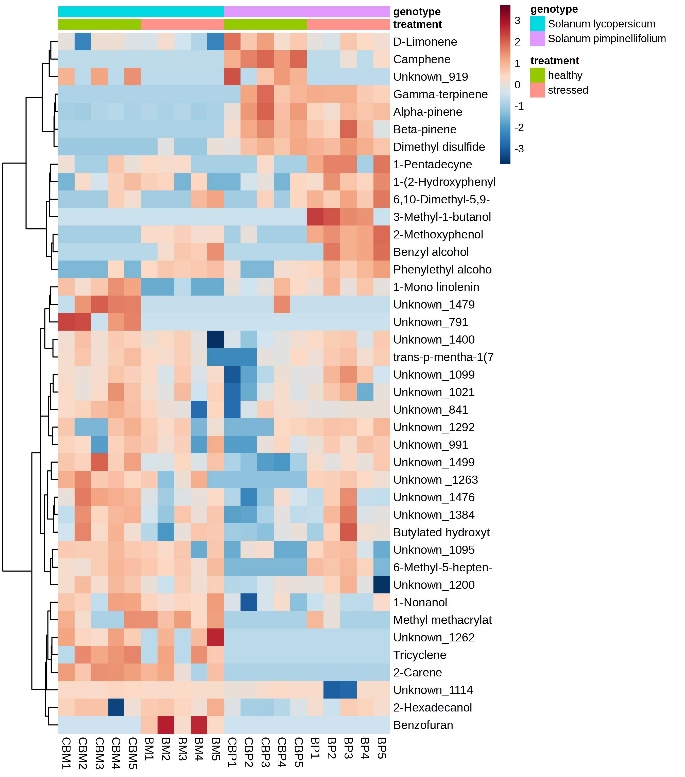


*****

*****

**Supplementary Figure S8.** **(A)** Root volatile profile of S. pimpinellifolium and S.lycpersicum cv Moneymaker (un)infected with Botrytis cinerea detected from an in-vitro set up. A heatmap showing a comparison of normalized peak areas (log-transformed and Pareto-scaled) of selected mass features across different treatments. Mass features were selected based on their pairwise comparisons across treatments (healthy S.lycopersicum (CBM), healthy S.pimpinellifolium (CBP), Botrytis-infected S.lycopersicum (BM) and Botrytis-infected S.pimpinellifolium (BP) using multiple testing correction (FDR). Only statistically significant features (FDR<0.05) in at least one comparison are in included in the heatmap. Clustering of the samples was performed using the Euclidean distance. The intensity of normalized peak area is indicated by the color gradient legend with darker blue color suggesting a lower intensity while the darker red color indicating the higher peak area intensity. The bottom column codes refer to different treatments with numbers refer to the number of replicate for each treatment. **(B)** Spearman rank correlation coefficients of the top 25 root-associated volatile compounds differentially detected upon Botrytis cinerea infection in tomato plants grown in the in-vitro system. Red bars and blue bars indicate compounds with positive and negative association with the infected plants, regardless of genotype. Benzyl alcohol and benzofuran are among the volatile compounds positively associated with the stress responses of tomato plants (regardless the genotype)s upon the infection of Botrytis cinerea. Some unidentified compounds are referred to unknowns followed by numbers indicating their calculated retention indices.


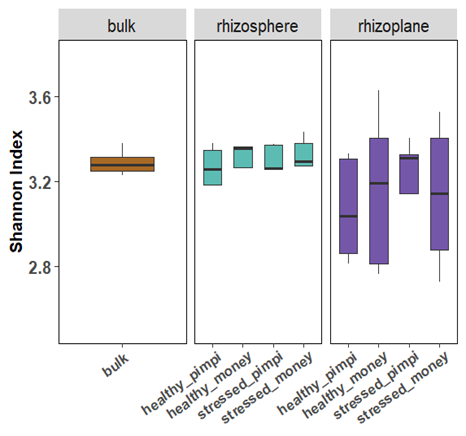

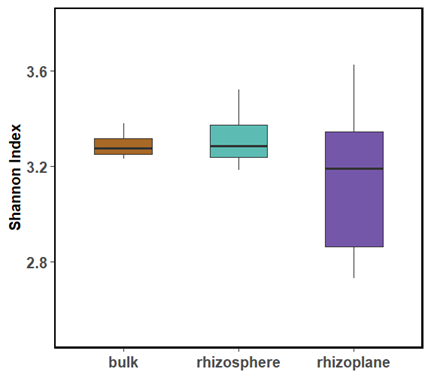

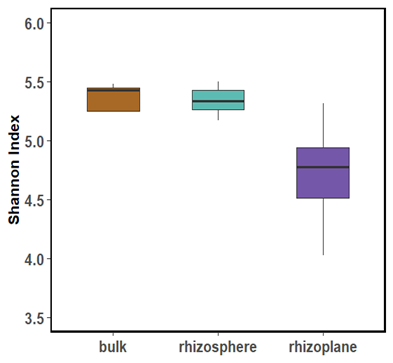

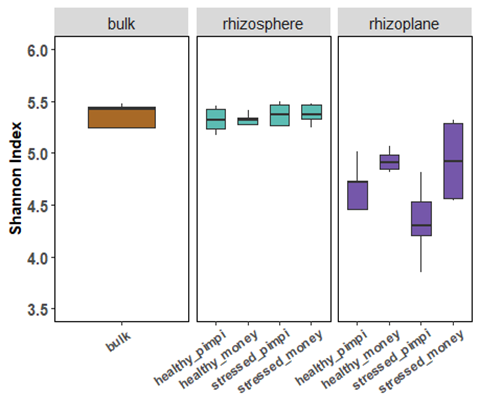


*****

**(B)**

**(A)**

**(D)**

**(C)**

**Supplementary Figure S9.** Boxplots showing alpha diversity of pooled datasets for bacterial **(A)** and fungal community **(B)** as estimated by the Shannon diversity index. The boxplots were then regrouped into an individual treatment within each compartment for bacterial **(C)** and fungal community **(D)**. Except the unplanted bulk soil (bulk), the rhizosphere and rhizoplane compartments contain four conditions; uninfected S.pimpinellifolium (healthy_pimpi, n=5 independent biological replicates), Botrytis-infected S.pimpinellifolium (stressed-pimpi, n=5 independent biological replicates), uninfected S.lycopersicum cv Moneymaker (healthy_money, n=5 independent biological replicates), and Botrytis-infected S.lycopersicum cv Moneymaker (stressed_money , n=5 independent biological replicates). The star (*) symbol indicated statistical difference (p<0.05).

**(A))**

**(B))**


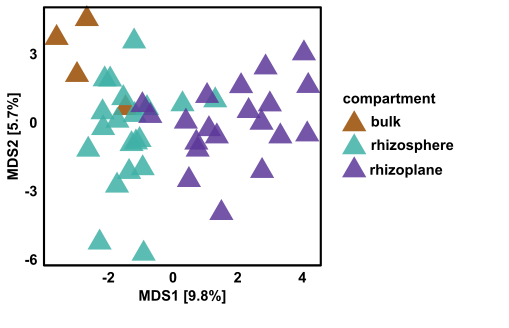

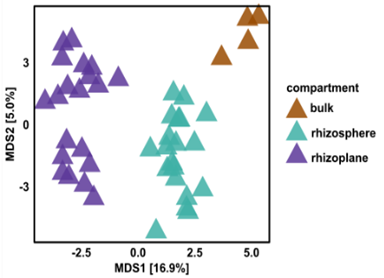


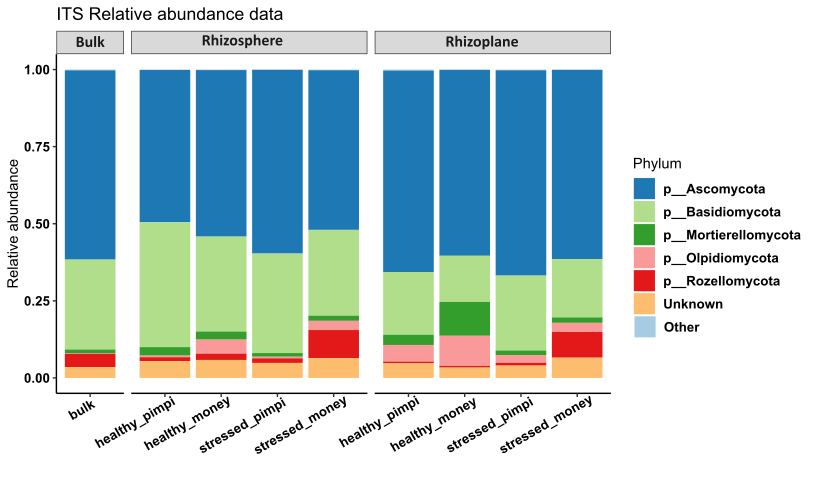

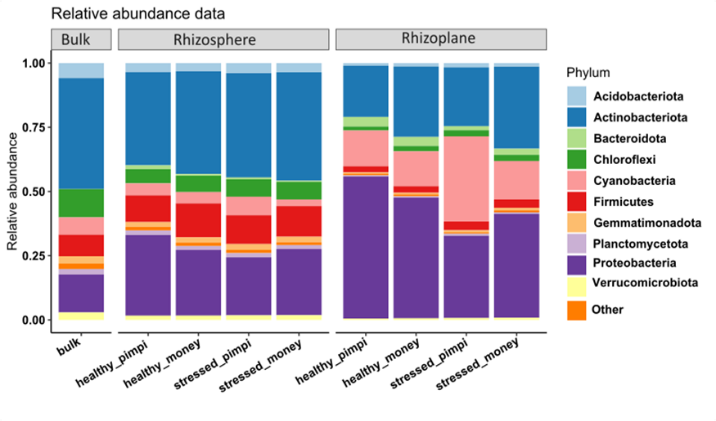
Z

**Supplementary Figure S10.** Principal coordinate analysis (PCoA) of bacterial **(A)** and fungal community **(B)** at the ASV level based on the Aitchison distance for the pooled dataset, including bulk soil. Clustering was observed between the three compartments; bulk soil (brown triangle), rhizosphere (green triangle), and rhizoplane (purple triangle). The stack bar plot showing the relative abundances of most dominant bacterial **(C)** and fungal **(D)** phyla from different compartments. For each compartment, except the unplanted bulk soil(bulk), contains four treatments; uninfected S.pimpinellifolium (healthy_pimpi, n=5 independent biological replicates), Botrytis-infected S.pimpinellifolium (stressed-pimpi, n=5 independent biological replicates), uninfected S.lycopersicum cv Moneymaker (healthy_money, n=5 independent biological replicates), and Botrytis-infected S.lycopersicum cv Moneymaker (stressed_money, n=5 independent biological replicates)

**(C))**

**(D))**


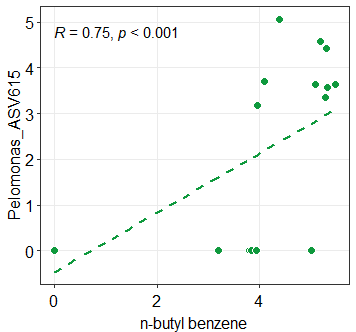

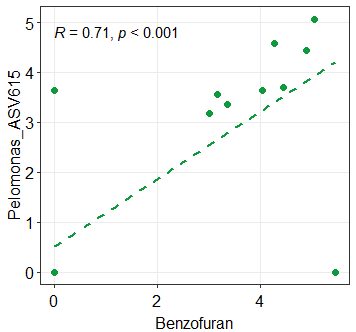

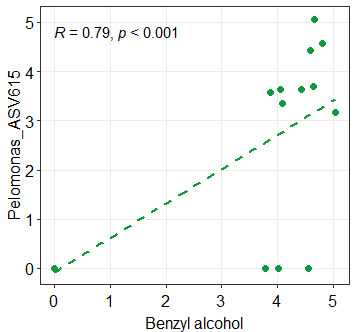

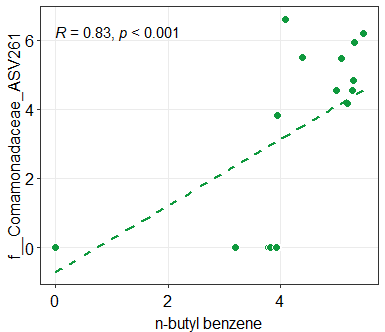

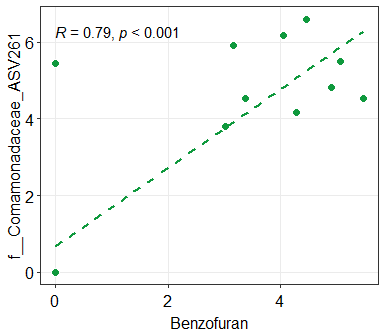

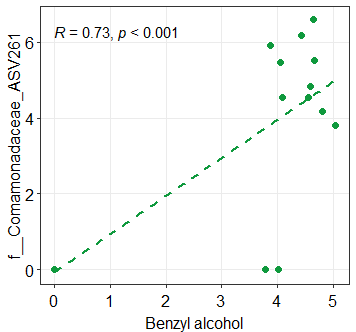


**Supplementary Figure S11**. Scatter plots showing Spearman’s correlation between rhizoplane bacterial taxa and root-associated volatile compounds. In y-axis, the relative abundances (%) of taxa enriched in rhizoplane of both tomato genotypes upon the foliar infection: Cylindrospermum (ASV61), f_Comamonadaceae (ASV261), and Pelomonas (ASV615) were positively correlated with stress-induced volatile compounds (normalized peak areas) in tomato plants; Benzyl alcohol, benzofuran and n-butyl benzene.
