# Supplementary material for: Foliar infections by Botrytis cinerea modulate the tomato root volatilome and microbiome
Source: FEMS Microbiol Ecol. 2025 Apr 18;101(5):fiaf042. doi: 10.1093/femsec/fiaf042 (PMC12023855; doi:10.1093/femsec/fiaf042)
Supplement: fiaf042_Supplemental_Files [file fiaf042_supplemental_files.zip › revised_Supplementary Table.docx]

**Supplementary Table S1.** Pairwise comparison (Deseq2 contrast) results of rhizosphere **bacterial ASVs** that are enriched (red) and depleted (green) as well as their average relative abundance (%) in the Botrytis-infected S.pimpinellifolium (compared to non-infected S.pimpinellifolium )

**Supplementary** **Table S2.** Pairwise comparison (Deseq2 contrast) results of **bacterial ASVs** that are enriched (red) and depleted (green) as well as their average relative abundance (%) in the rhizosphere of Botrytis-infected S.lycopersicum var. Moneymaker (compared to non-infected S.lycopersicum var. Moneymaker )

**Supplementary** **Table 3.** Pairwise comparison (Deseq2 contrast) results of **fungal ASVs** that are enriched (red) and depleted (green) as well as their average relative abundance (%) in the rhizosphere of Botrytis-infected S.pimpinellifolium (compared to non-infected S.pimpinellifolium )

**Supplementary Table S4.** Pairwise comparison (Deseq2 contrast) results of **fungal ASVs** that are enriched (red) and depleted (green) as well as their average relative abundance (%) in the rhizosphere of Botrytis-infected S.lycopersicum var. Moneymaker (compared to non-infected S.lycopersicum var. Moneymaker )

**Supplementary Table S5**. Pairwise comparison (Deseq2 contrast) results of **bacterial ASVs** that are enriched (red) and depleted (green) as well as their average relative abundance (%) in the rhizoplane of Botrytis-infected S.pimpinellifolium (compared to non-infected S.pimpinellifolium )

**Supplementary Table S6.** Pairwise comparison (Deseq2 contrast) results of **bacterial ASVs** that are enriched (red) and depleted (green) as well as their average relative abundance (%) in the rhizoplane of Botrytis-infected S.lycopersicum var. Moneymaker (compared to non-infected S.lycopersicum var. Moneymaker)

**Supplementary Table S7.** Pairwise comparison (Deseq2 contrast) results of **fungal ASVs** that are enriched (red) and depleted (green) as well as their average relative abundance (%) in the rhizoplane of Botrytis-infected S.pimpinellifolium (compared to non-infected S.pimpinellifolium )

**Supplementary Table S8**. Pairwise comparison (Deseq2 contrast) results of fungal ASVs that are enriched (red) and depleted (green) as well as their average relative abundance (%) in the rhizoplane of Botrytis-infected S.lycopersicum var. Moneymaker (compared to non-infected S.lycopersicum var. Moneymaker)
